# DONSON and FANCM associate with different replisomes distinguished by replication timing and chromatin domain

**DOI:** 10.1038/s41467-020-17449-1

**Published:** 2020-08-07

**Authors:** Jing Zhang, Marina A. Bellani, Ryan C. James, Durga Pokharel, Yongqing Zhang, John J. Reynolds, Gavin S. McNee, Andrew P. Jackson, Grant S. Stewart, Michael M. Seidman

**Affiliations:** 1 Laboratory of Molecular Gerontology, National Institute on Aging, National Institutes of Health, Baltimore, MD 21224 USA; 2 Department of Molecular Biology and Genetics, Cornell University, Ithaca, NY 14850 USA; 3 Horizon Discovery, Lafayette, CO 80026 USA; 4 Gene Expression and Genomics Unit, National Institute on Aging, National Institutes of Health, Baltimore, MD 21224 USA; 5 Institute of Cancer and Genomic Sciences, College of Medical and Dental Sciences, University of Birmingham, Birmingham, UK; 6 MRC Human Genetics Unit, Institute of Genetics and Molecular Medicine, University of Edinburgh, Edinburgh, UK

**Keywords:** Chromosomes, Replisome

## Abstract

Duplication of mammalian genomes requires replisomes to overcome numerous impediments during passage through open (eu) and condensed (hetero) chromatin. Typically, studies of replication stress characterize mixed populations of challenged and unchallenged replication forks, averaged across S phase, and model a single species of “stressed” replisome. Here, in cells containing potent obstacles to replication, we find two different lesion proximal replisomes. One is bound by the DONSON protein and is more frequent in early S phase, in regions marked by euchromatin. The other interacts with the FANCM DNA translocase, is more prominent in late S phase, and favors heterochromatin. The two forms can also be detected in unstressed cells. ChIP-seq of DNA associated with DONSON or FANCM confirms the bias of the former towards regions that replicate early and the skew of the latter towards regions that replicate late.

## Abstract (web summary)

Eukaryotic replisomes are multiprotein complexes. Here the authors reveal two distinct stressed replisomes, associated with DONSON and FANCM, displaying a bias in replication timing and chromatin domain.

## Introduction

Eukaryotic replisomes are multiprotein complexes consisting, minimally, of the CMG helicase [MCM2-7 (M), CDC45 (C), and GINS (go, ichi, ni, san) proteins (G)], which forms a ring around the leading strand template. Other components include the pol α, ε, and δ polymerases, MCM10, and a few accessory factors^1–7^. The identification and characterization of the minimal components of biochemically active replisomes, the result of decades of extraordinary work from multiple laboratories, necessarily reflects studies with deproteinized model DNA substrates under carefully controlled conditions. However, in vivo there are hundreds of replisome-associated proteins^8,9^. Presumably, this reflects the multiple layers of complexity that characterize replication of the genome in living cells. For example, three-dimensional analyses of chromosome structure demonstrate two major domains. The A compartment contains euchromatin, which is accessible, transcriptionally active, and marked by specific histone modifications, such as H3K4me3. The B compartment, which is more condensed, contains inactive genes, many repeated elements, and is associated with different histone modifications, including H3K9me3^10^. In addition to the structural distinctions, regions of the genome are also subject to temporal control of replication during the S phase. Sequences in Compartment A tend to replicate early in the S phase, while those in B are duplicated in late S phase^11,12^.

Other influential effectors of replisome composition are the frequent encounters with impediments, that stall or block either the progress of the CMG helicase or DNA synthesis. These include alternate DNA structures, protein: DNA adducts, DNA covalent modifications introduced by exogenous or endogenous reactants, depleted nucleotide precursor pools, etc. Replication stress activates the ATR (ATM- and Rad3-related) kinase, with hundreds of substrates, including MCM proteins^13,14^, and stimulates the recruitment of numerous factors to stalled replication forks^15,16^. These function in a variety of pathways to relieve obstacles, reconstruct broken forks, and restart replication.

We have developed an approach to study replication stress imposed by an interstrand cross-link (ICL). While these have always been considered absolute blocks to any process requiring DNA unwinding^17,18^, we found that replication could restart (traverse) past an intact ICL in the genome of living cells^19^ (see also ref. ^20^). Traverse of the ICL was dependent on ATR, and, in part, on the translocase activity of FANCM^21,22^. FANCM was recruited to ICL-proximal replisomes, which were marked by phosphorylation of MCM2 by ATR. Furthermore, the association with FANCM was accompanied by remodeling of replisomes characterized by the loss of the GINS complex^23^.

The partial dependence of ICL traverse on FANCM raised the question of what other factor(s) would support this activity. Recently, the DONSON protein was described as mutated in a microcephalic dwarfism syndrome^24,25^. This essential protein, which has no recognizable structural features, associates with replisomes and contributes to the response to replication stress. In the work described here, we find that, like FANCM, DONSON is complexed with ICL-proximal replisomes also lacking the GINS proteins. The two stressed replisomes are distinguished by activity in different stages of S phase and different chromatin regions. In cells without ICLs, DONSON and FANCM associate with sequences that show the same differential biases in replication timing and chromatin domain.

## Results

### DONSON contributes to ICL traverse

**Fig. 1:**
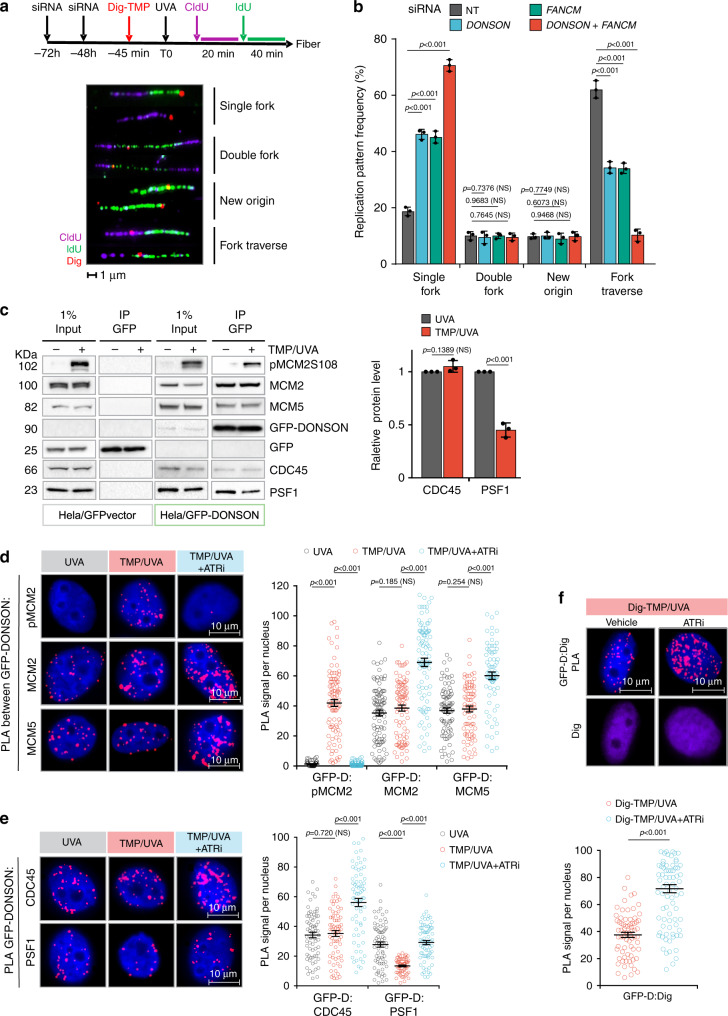
DONSON and FANCM operate in separate pathways to promote replication traverse. **a** HeLa cells were treated with siRNA against DONSON or FANCM or both. They were exposed to Dig-TMP/UVA and incubated with CldU, then IdU. Fibers were prepared and patterns displayed by immunofluorescence against the analogues and immunoquantum detection (Q dot 655, in red) for Dig-tagged ICLs. Representative patterns are shown. **b** Quantitation of pattern distribution. Fibers with ICL encounters: NT = 417; siDONSON = 432; siFANCM = 417; siDONSON + siFANCM = 385, from three independent replicates. Data are mean ± s.d. **c** IP immunoblot of chromatin proteins from cells expressing GFP (panels 1, 2) or GFP-DONSON (panels 3, 4) exposed to UVA (−) or TMP/UVA (+). The identity of the proteins is indicated. The amounts of PSF1 and CDC45 in the two samples were quantitated. Representative blot (*n* = 3). Data are mean ± s.d. **d** PLA test of the influence of ATR inhibition on GFP-DONSON interactions with pMCM2S108, MCM2, and MCM5. Number of nuclei: PLA between GFP-DONSON and pMCM2 in cells treated with UVA = 58, TMP/UVA = 94, TMP/UVA + ATRi = 55; PLA between GFP-DONSON and MCM2 in UVA = 95, TMP/UVA = 89, TMP/UVA + ATRi = 93; PLA between GFP-DONSON and MCM5 in UVA = 88, TMP/UVA = 79, TMP/UVA+ATRi = 73; from three biological replicates. Data are mean ± s.e.m. **e** PLA assessing the influence of ATR inhibition on GFP-DONSON interactions with CDC45 and PSF1. Scored nuclei of PLA between GFP-DONSON and CDC45 in UVA = 70, TMP/UVA = 71, TMP/UVA + ATRi = 73; scored nuclei of PLA between GFP-DONSON and PSF1 in UVA = 71, TMP/UVA = 64, TMP/UVA + ATRi = 77; from three biological replicates. Data are mean ± s.e.m. **f** Influence of ATR inhibition on the PLA between GFP-DONSON and Dig-tagged ICLs. Scored nuclei: Vehicle = 72, ATRi = 87, three biological replicates. Data are mean ± s.e.m. For replication pattern frequency experiments and Western blotting image analysis (**a**, **c**), a two-sided unpaired *t* test was used to calculate *P*-values. For PLA experiments (**d**–**f**), a two-sided Mann–Whitney rank-sum test was used to determine if differences were significant. NS: not significant: *P* > 0.05. Source data are provided as a Source Data file.

We have developed an approach to follow replication in the vicinity of antigen-tagged ICLs. Cells were treated with digoxigenin–trimethylpsoralen and long-wave ultraviolet light (Dig-TMP/UVA) and pulsed sequentially with 5-chloro-2′-deoxyuridine (CldU) and 5-iodo-2′-deoxyuridine (IdU) prior to spreading DNA fibers. Staining of the incorporated analogues and the Dig tag displays the outcomes of fork encounters with ICLs (Fig. 1a; Supplementary Fig. 1a, b). Replication restart past ICLs (traverse) was reduced in cells deficient for FANCM, as shown previously^19^. Recently, the DONSON protein was shown to contribute to the cellular response to replication stress^24,25^. While DONSON does not appear to be a conventional DNA repair factor (it was not important for survival of cells exposed to cisplatin, Supplementary Fig. 1c), reduced expression of DONSON, either by siRNA knockdown (Fig. 1b; Supplementary Fig. 1d), or by mutation in patient-derived cells (Supplementary Fig. 1e), did influence the results of the replication/fiber assay. Traverse frequency was reduced in these cells and declined further in doubly deficient cells, indicating that DONSON and FANCM were non epistatic for ICL traverse (Fig. 1b).

A relationship with replication and the replisome was indicated by co-immunoprecipitation of the endogenous DONSON protein or a GFP-tagged DONSON with MCM proteins from untreated cells (no TMP/UVA) consistent with the prior report^24^. DONSON was also complexed with CDC45 and the GINS proteins indicating association with the helicase functional form of the replisome (Supplementary Fig. 1f), in contrast to replisomes bound by FANCM^23^. Proximity ligation assays (PLA)^26,27^ confirmed these interactions (Supplementary Fig. 1g). After TMP/UVA treatment, the association with MCM proteins and CDC45 was maintained while the interaction with PSF1, a GINS protein, was reduced (Fig. 1c). In the treated cells, PLA reported the proximity of DONSON and MCM proteins and also pMCM2S108, phosphorylated by ATR at S108 (Fig. 1d). The PLA between DONSON and PSF1 was positive in control cells and reduced in TMP/UVA cells (Fig. 1e) in agreement with the IP. These data demonstrated the association of DONSON with replisomes in cells with or without TMP/UVA treatment. Furthermore, they distinguished DONSON from FANCM, which, as shown previously, was not in complex with GINS proteins in either condition^23^.

Inhibition of ATR blocks the association of FANCM with replisomes^23^. In contrast, the PLA between DONSON and the ICLs was positive in control cells, and increased after ATR inhibition (Fig. 1f). Thus, the response to ATR inhibition also differentiated the DONSON: replisome from the FANCM: replisome. These results are explained by a scenario in which encounters of DONSON: replisomes with ICLs are accompanied by the loss of GINS and traverse of the ICL. In the presence of the ATR inhibitor those replisomes accumulate at ICLs, traverse is blocked, and the GINS retained.

### DONSON and FANCM are on different replisomes

**Fig. 2:**
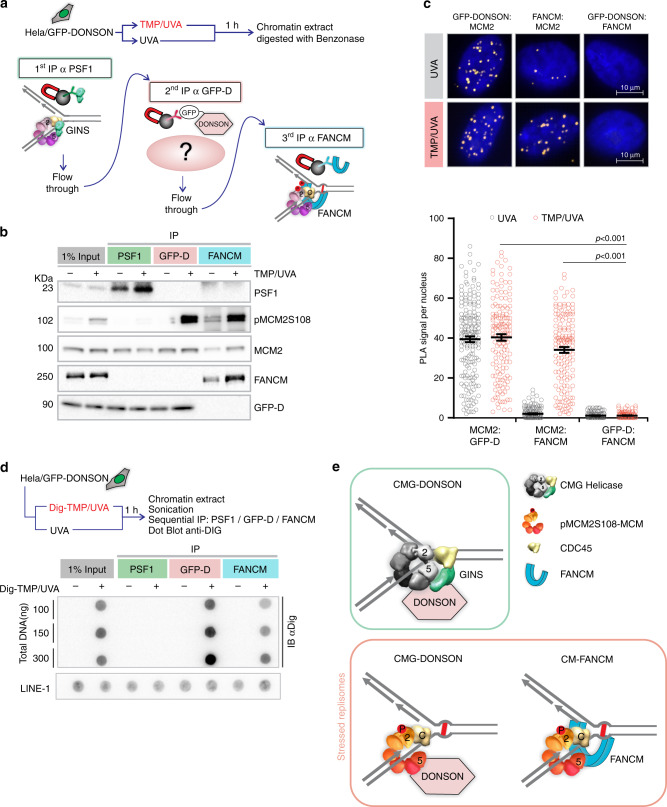
DONSON and FANCM are on different replisomes. **a** Scheme of sequential IP against DONSON and FANCM-associated replisomes. HeLa cells expressing GFP-DONSON were exposed to UVA only or TMP/UVA. Chromatin was prepared and digested with benzonase. This was followed by IP against PSF1 (to remove unstressed replisomes), then IP of the supernatant against GFP (to remove remaining DONSON-associated proteins), and finally IP of the residual supernatant to capture FANCM-bound proteins. **b** Western blot analysis of sequential IP. Representative blot (*n* = 3). **c** PLA in cells exposed to UVA only or TMP/UVA shows interactions between GFP-DONSON and MCM2; and FANCM and MCM2; but not between GFP-DONSON and FANCM. Scored nuclei: PLA between GFP-D: MCM2, UVA treatment = 174; TMP/UVA = 148; PLA between FANCM: MCM2, UVA = 142; TMP/UVA = 145; PLA between GFP-D: FANCM, UVA = 135; TMP/UVA = 133 from three biological replicates. Data are mean ± s.e.m. A two-sided Mann–Whitney rank-sum test was used to determine if differences were significant. NS: not significant: *P* > 0.05. **d** Association of replisomes with Dig-tagged ICLs. Chromatin was prepared from cells exposed to UVA or Dig-TMP/UVA, and the DNA reduced to fragments of <500 bp by sonication. Sequential IP was performed, and the DNA isolated from each fraction, dotted onto the nitrocellulose, and probed with an antibody to the Dig tag. LINE-1 repeat element served as a loading control. Representative blot (*n* = 2). **e** Model summarizing the results of the sequential IP experiment. Source data are provided as a Source Data file.

To determine if FANCM and DONSON were on the same replisomes, we performed a sequential immunoprecipitation (IP) experiment (Fig. 2a). Chromatin was prepared from GFP-DONSON cells exposed to TMP/UVA, the DNA digested, and protein complexes incubated with antibody against PSF1, which served as a marker of a fully functional, nonstressed, replisome. The precipitate contained the target PSF1, MCM2, DONSON, but neither FANCM nor pMCM2S108 (Fig. 2b). A second cycle of IP confirmed clearance of these replisomes (Supplementary Fig. 2a). The supernatant was then incubated with antibody against GFP-DONSON. The IP contained GFP-DONSON and pMCM2S108, but no FANCM and, as expected, no PSF1. After another IP against GFP-DONSON, the remaining supernatant was incubated with antibody against FANCM. This IP contained FANCM, pMCM2S108, but no DONSON and no PSF1. Reversal of the order of the IP (FANCM before DONSON) did not change the results (Supplementary Fig. 2b). Thus, there were two DONSON-associated replisomes: (1) replisome: CMG-D, independent of TMP/UVA, not marked by ATR phosphorylation, associated with the GINS; (2) replisome: CM-D, induced by TMP/UVA, with pMCM2S108, but not PSF1 or FANCM. The FANCM complex, replisome: CM-F, had pMCM2S108, but no GINS or DONSON. CDC45 and the auxiliary proteins, MCM10, MCM8, and RAD51, were found in all samples (Supplementary Fig. 2c). These experiments were performed in HeLa cells expressing GFP-DONSON. In order to test the generality of these results, we repeated the experiment in the hTERT immortalized diploid RPE1 cell line derived from retinal pigment epithelial cells and used in many studies of the cellular response to genotoxic stress^28^. They displayed the same high frequency of ICL traverse as the HeLa and DONSON complemented patient-derived cells (Supplementary Fig. 2d). The serial IP was performed, except that the antibody against the endogenous DONSON protein was employed. The results were identical to those with the GFP-DONSON HeLa cells (Supplementary Fig. 2e).

PLA analyses with the GFP-DONSON HeLa cells agreed with the IP experiments. The interaction of DONSON with MCM2 in both UVA and TMP/UVA-treated cells was positive (Fig. 2c). The PLA between FANCM and MCM2, which was detectable but low in cells without ICLs, was greatly increased in cells treated with TMP/UVA, while the PLA between DONSON and FANCM was negative in control and TMP/UVA-treated cells.

We then tested the replisomes for association with ICLs. We treated cells with Dig-TMP/UVA and performed a sequential immunoprecipitation on chromatin sonicated to small DNA fragment size (sequential ChIP) in the order as in Fig. 2a. The DNA from each IP was recovered and examined for the presence of the Dig tag. There was no signal in the PSF1 sample, but both the subsequent precipitates were positive. Consequently, the ICLs were associated with replisomes containing CM-DONSON and CM-FANCM, but not CMG-DONSON (Fig. 2d, e; Supplementary Fig. 2f).

### DONSON and FANCM replisomes at early and late S phase

**Fig. 3:**
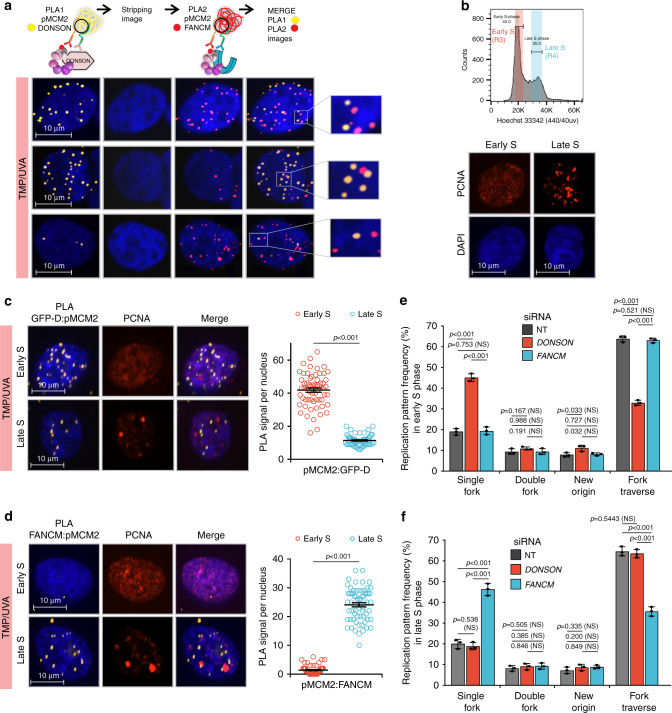
DONSON and FANCM replisomes are active in different stages of the S phase. **a** Analysis by sequential PLA of GFP-DONSON: pMCM2S108 complexes and then FANCM: pMCM2S108, in GFP-DONSON expressing cells exposed to TMP/UVA. After the first PLA, the cells were photographed (first column of images) and the antibodies and PLA product stripped (second column). The second PLA was performed, and the cells re-imaged (third column). The fourth column shows a merge of both images after image registration in the *xyz* planes using the DAPI signal. Examples are shown of cells with strong signals from both first and second PLA, or strong signals from the first but infrequent from the second, or weak from the first and strong from the second. The signals from the two PLA do not colocalize. **b** Early and late S phase fractions were isolated from sorted cells. The PCNA staining pattern from each fraction. **c** GFP-D: pMCM2S108 PLA in sorted early and late S phase cells. Scored nuclei: GFP-D: pMCM2S108 of early S phase = 62, late S phase = 60, from three biological replicates. Data are mean ± s.e.m. **d** FANCM: pMCM2S108 PLA in sorted early and late S phase cells. Scored nuclei: FANCM: pMCM2S108 of early S phase = 63, late S phase = 64, from three biological replicates. Data are mean ± s.e.m. **e**, **f** Influence of DONSON and FANCM on patterns of replication encounters with ICLs in early and late S phase cells. Cells were treated with siRNA against DONSON or FANCM, exposed to Dig-TMP/UVA and pulsed with nucleoside analogues as in Fig. 1a. Cells were sorted, and fiber patterns from early and late S phase analyzed. Data are mean ± s.d.. For PLA experiments (**c**, **d**), a two-sided Mann–Whitney rank-sum test was used, for replication pattern experiments (**e**, **f**) a two-sided unpaired *t* test was used, to determine if differences were statistically significant. NS: not significant: *P* > 0.05. Source data are provided as a Source Data file.

To determine if CM-DONSON and CM-FANCM replisomes were in the same cell at the same time, we performed sequential PLA on TMP/UVA-treated cells grown on plates marked to facilitate re analysis of the same cells (Fig. 3a; Supplementary Fig. 3a). Images were taken of the DONSON: pMCM2S108 PLA, the plates were stripped of antibodies, followed by FANCM: pMCM2S108 PLA. The cells examined in the first analysis were re-imaged, and the two images aligned in *x*, *y*, *z* (Methods). Some cells had more DONSON: pMCM2S108 signals than FANCM: pMCM2S108, while the opposite was true for others (Fig. 3a). Furthermore, although some cells had signals from both assays, they did not colocalize, indicating that these replisomes were in different genomic locations (Supplementary Movie 1).

In an effort to understand the basis of these results, we treated cells with TMP/UVA and then recovered early and late S phase cells by flow cytometry (Fig. 3b; Supplementary Fig. 3b). The PLA between the Dig-tagged ICLs and pS108MCM2 showed equal frequencies of ICL-proximal stressed replisomes in the two cell fractions (Supplementary Fig. 3c). DONSON: pMCM2S108 and FANCM: pMCM2S108 PLAs were performed on each group. The DONSON complex was about fourfold more frequent in early S phase than in late, while the FANCM complex was about tenfold more frequent in late S phase than in early (Fig. 3c, d). The negative PLA for both partner sets in the G_1_ phase cells provided an important internal control for the specificity of the reagents and assay (Supplementary Fig. 3d).

The clear distinction between the early and late S phase fractions reflected the separation of the early S phase cells from those in late S phase. On the other hand, when we examined mid S phase cells, the pronounced difference between the PLA frequencies of the two stressed replisomes was lost, indicating that both stressed replisomes were present in mid S phase cells (Supplementary Fig. 3e).

The influence of DONSON on replication fork encounters with ICLs in the early and late stages of S phase was tested in cells treated with siRNA/DONSON. There was an increase in single-fork stalling events and a decline in traverse frequency in the early S phase cells, while there was little change in late S phase cells (Fig. 3e, f). Conversely, in cells treated with siRNA/FANCM, there was an increase in single-fork stalling and a decline in traverse frequency in late S phase cells, with relatively little effect on early S phase patterns (Fig. 3e, f). Thus, the DONSON: stressed replisome made a greater contribution to the traverse patterns in early S phase than in late, while the FANCM: replisome was more important in late S phase. Consequently, deficiencies in one or the other would differentially influence the outcome of replisome encounters with ICLs depending on the stage of the S phase.

**Fig. 4:**
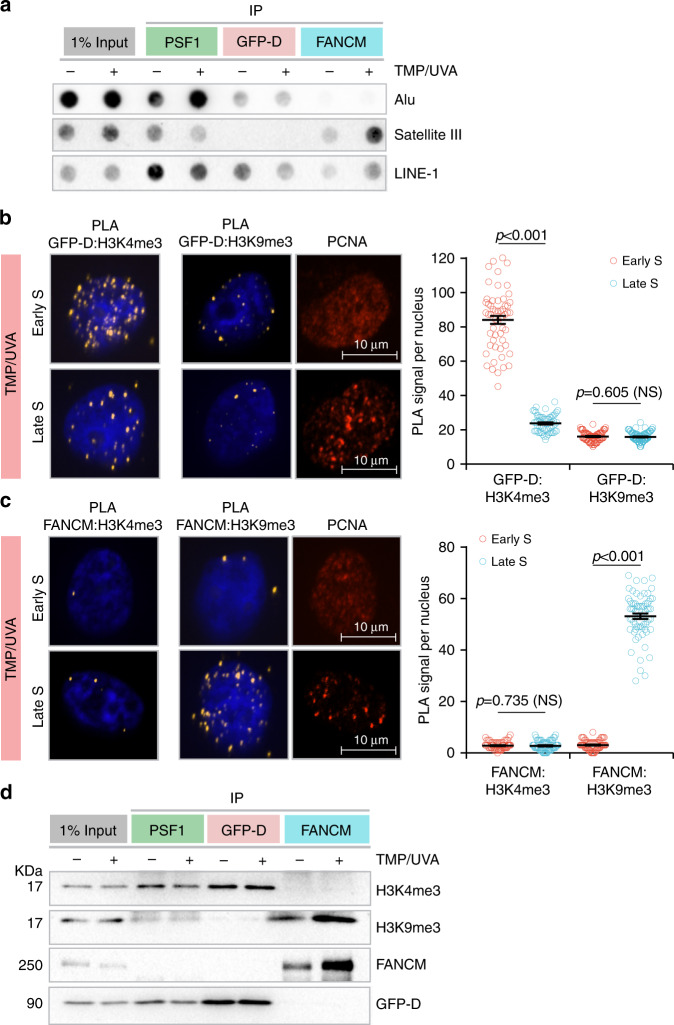
Relationship of DONSON and FANCM to replication timing and chromatin domain. Cells were treated with either UVA or TMP/UVA. **a** Sequential IP demonstrates association of early-replicating Alu sequences with DONSON and late-replicating Satellite 3 sequences with FANCM. LINE-1 elements replicate throughout the S phase and are found in all fractions. Representative blot (*n* = 3). **b** DONSON interaction with the H3K4me3 euchromatin mark is more frequent in early S phase cells than in late S phase, while there is little interaction with the H3K9me3 heterochromatin mark in either stage. Sorted early and late S phase cells were examined by PLA. Scored nuclei: PLA between GFP-D: H3K4me3 of early S phase = 67, late S phase = 64, PLA between GFP-D: H3K9me3 of early S phase = 70, late S phase = 85, from three biological replicates. Data are mean ± s.e.m. **c** FANCM interaction with H3K9me3 heterochromatin mark is biased toward late S phase, while there is low interaction frequency with H3K4me3 in either stage. Scored nuclei: PLA between FANCM: H3K4me3 of early S phase = 64, late S phase = 66, PLA between FANCM: H3K9me3 of early S phase = 67, late S phase = 77, from three biological replicates. Data are mean ± s.e.m. **d** Sequential IP demonstrates greater association of DONSON with H3K4me3 than H3K9me3 and greater association of FANCM with H3K9me3 than H3K4me3. Representative blot (*n* = 3). For PLA experiments in **b**, **c**, a two-sided Mann–Whitney rank-sum test was used to determine if differences were statistically significant. NS: not significant: *P* > 0.05. Source data are provided as a Source Data file.

Alu sequences are replicated in early S phase, Satellite 3 sequences are replicated in late S phase, while LINE-1 elements are replicated throughout^29^. Cells were treated with TMP/UVA and DNA isolated from each fraction from the sequential ChIP (as in Fig. 2f) and examined for the presence of these repeats. As expected, the replisome marked by PSF1 was associated with all the sequences. However, the recovery of Alu sequences was biased toward the replisome: CM-DONSON fraction, while the recovery of Satellite 3 was greater with the replisome: CM-FANCM (Fig. 4a). LINE-1, which replicates throughout the S phase, was found in all fractions.

Active genes replicate in early S phase, and are found in euchromatin, marked by histone H3K4 trimethylation^30^, while inactive genes replicate late, and are in heterochromatin, characterized by H3K9 trimethylation^31^. We treated cells with TMP/UVA and examined the proximity of GFP-DONSON to the two chromatin marks in early and late S phase cells. The PLA with H3K4me3 showed a fourfold higher signal frequency in early S phase than in late, while the PLA with H3K9me3 was much weaker in both stages (Fig. 4b). The PLA between FANCM and H3K4me3 was quite low in both early and late S phase cells, while the signal with H3K9me3 was about tenfold stronger in late S phase than in early S phase cells (Fig. 4c). These experiments were repeated in RPE1 cells with identical results (Supplementary Fig. 4a, b). They were also confirmed by sequential chromatin IP in both cell lines which showed that H3K4me3 was associated with the replisome: CM-DONSON complex, while the replisome: CM-FANCM was associated with H3K9me3 (Fig. 4d; Supplementary Fig. 4c). The results of these experiments confirmed the appearance of replisomes differing by association with either FANCM or DONSON in cells exposed to replication stress imposed by the ICLs.

### DONSON and FANCM replisomes in untreated cells

**Fig. 5:**
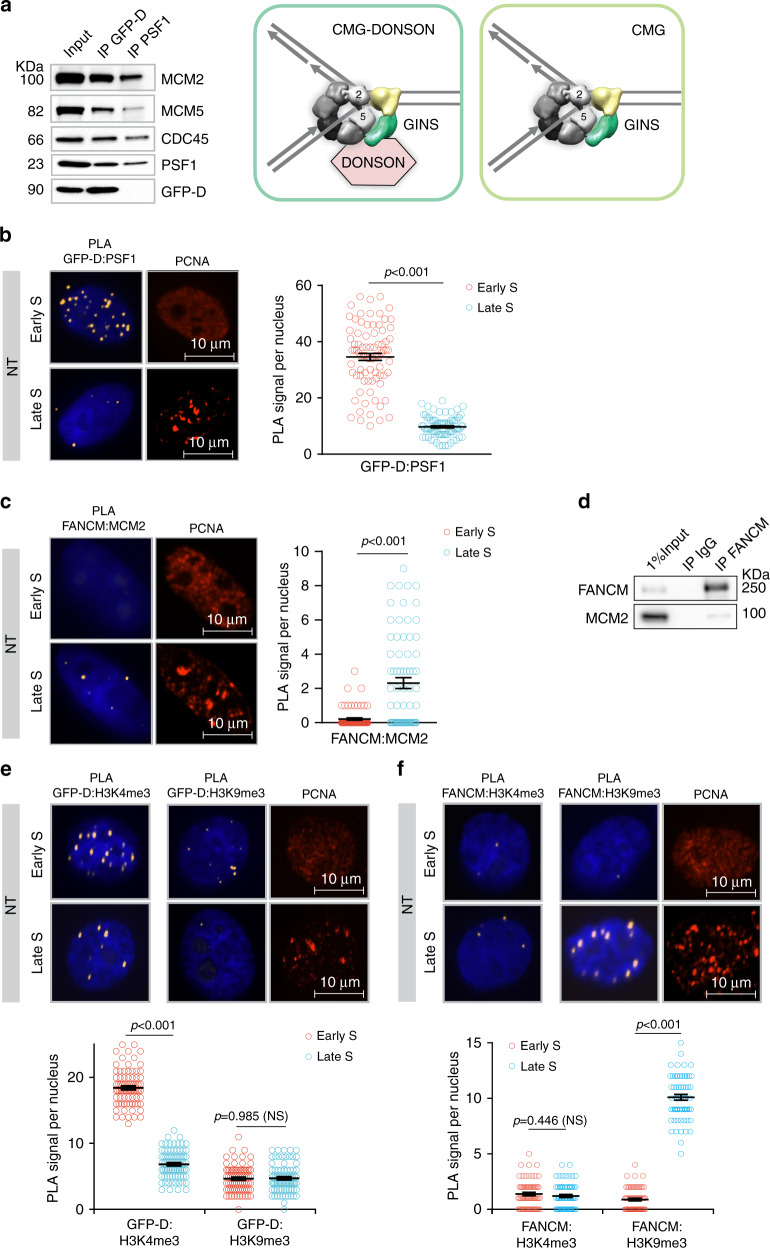
Interactions of DONSON and FANCM with replisomes and chromatin in nontreated cells. **a** DONSON associates with some, but not all, replisomes in untreated cells. Chromatin was prepared from untreated GFP-DONSON-HeLa cells and sequential IP performed, first against GFP-DONSON, and then against the GINS protein PSF1 from the residual supernatant. Representative blot (*n* = 3). **b** PLA of GFP-DONSON and PSF1 demonstrates DONSON-associated replisomes are more frequent in early S phase than in late S phase in NT cells. Scored nuclei: PLA between GFP-D: PSF1 early S phase = 82, late S phase = 81, from three biological replicates. Data are mean ± s.e.m. **c** PLA between FANCM and MCM2 demonstrates low level of FANCM-associated replisomes in late S phase in nontreated cells. Scored nuclei: PLA between FANCM: MCM2 of early S phase = 73, late S phase = 75, from three biological replicates. Data are mean ± s.e.m. **d** IP of FANCM demonstrates low-level interaction with replisome protein MCM2. **e** PLA between GFP-DONSON and H3K4me3 or H3K9me3. Scored nuclei of GFP-DONSON and H3K4me3 in early S phase = 79, late S phase = 78. Scored nuclei of GFP-DONSON and H3K9me3 in early S phase = 77, late S phase = 82, from three biological replicates. Data are mean ± s.e.m. **f** PLA between FANCM and H3K4me3 or H3K9me3. Scored nuclei of FANCM and H3K4me3 in early S phase = 64, late S phase = 65. Scored nuclei of FANCM and H3K9me3 in early S phase = 68, late S phase =  65, from three biological replicates. Data are mean ± s.e.m. For PLA experiments in **b**, **c**, **e**, **f**, a two-sided Mann–Whitney rank-sum test was used to determine if differences were statistically significant. NS: not significant: *P* > 0.05. Source data are provided as a Source Data file.

The preceding experiments characterized replisomes in cells containing ICLs and demonstrated the bias of DONSON replisomes toward early S phase. Previously, DONSON was shown to be bound to replisomes in cells without exposure to a DNA reactive compound^24^, leaving open the question of whether it was complexed with all replisomes, or only a subset. To address this, chromatin proteins from untreated cells were subjected to sequential IP, first with DONSON as the target, after which the supernatant was incubated with antibody against PSF1 to recover remaining functional replisomes. Two complexes were recovered: replisome: CMG-DONSON and, subsequently, replisome: CMG (Fig. 5a). These results identified two forms of the replisome in unstressed cells: one with DONSON and one without. We then asked if DONSON replisomes in untreated cells were more or less abundant in different stages of the S phase. The PLA between GFP-DONSON and PSF1 showed about a 3.5-fold bias toward the early S phase (Fig. 5b). The proximity of FANCM to MCM2, albeit at quite low frequency (Figs. 2c and  5c), was biased to late S phase in nontreated cells (Fig. 5c). The low frequency interaction of FANCM with replisome proteins was also observed by immunoprecipitation (Fig. 5d).

We also asked about the proximity of DONSON and FANCM to modified histones in untreated cells. Cells were sorted and examined by PLA between GFP-DONSON or FANCM and H3K4me3 or H3K9me3. The DONSON: H3K4me3 signals were distributed throughout the nuclei and were about threefold more frequent in early S phase than in late, while there was little signal with H3K9me3 in either stage (Fig. 5e). There was minimal association between FANCM and H3K4me3 in either stage, while the interaction with H3K9me3 was weak in early S phase but about tenfold stronger in late S phase (Fig. 5f). The FANCM: H3K9me3 PLA signals were largely localized on the nuclear periphery, reflecting the association of H3K9me3 chromatin with nuclear lamina^32^. Thus, DONSON and FANCM were largely resident in different chromatin domains without requirement for ICL-induced replication stress. Similar results were acquired with RPE1 cells (Supplementary Fig. 5a, b).

### Association of DONSON and FANCM with genomic sequences

**Fig. 6:**
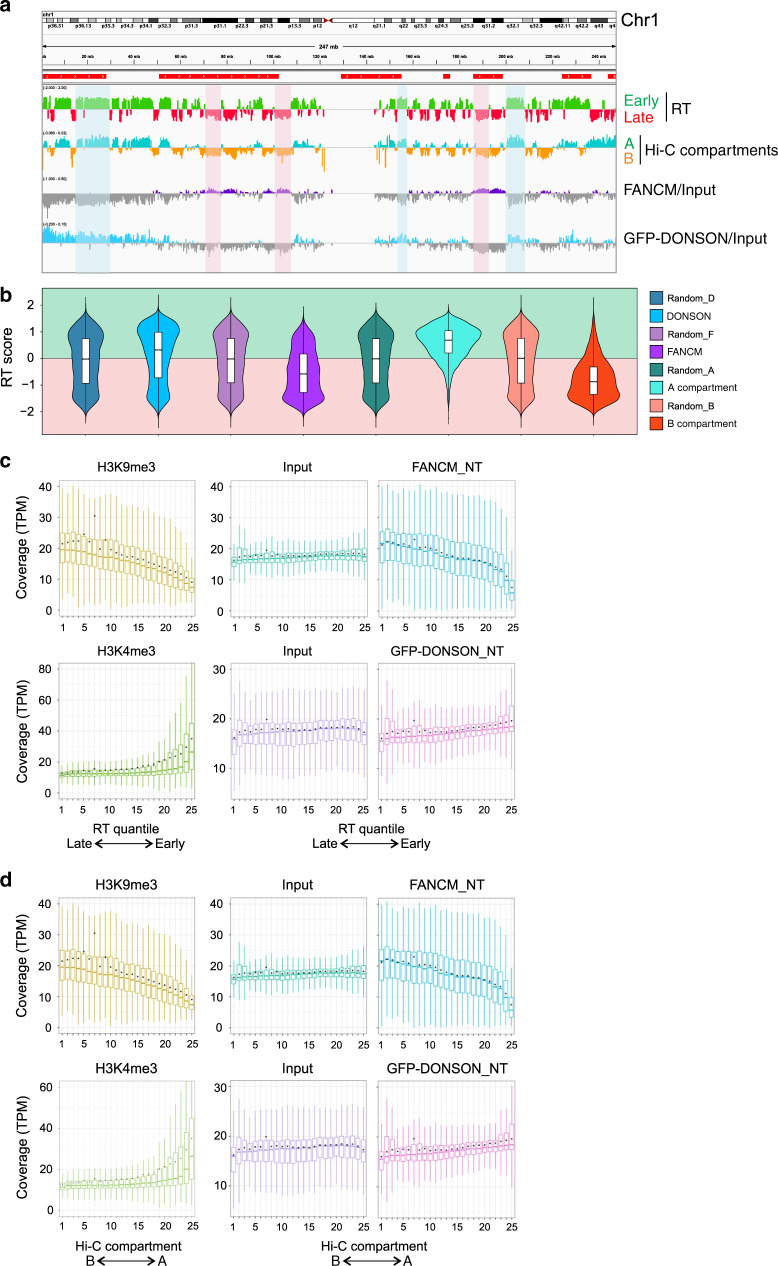
ChIP-Seq analysis of genome-wide distribution of FANCM and GFP-DONSON. **a** Representative profile from chromosome 1, comparing RT (RT = log2(Early/Late)) in HeLa cells, A/B compartments as defined by the eigenvector calculated from Hi-C data from HeLa cells, and FANCM and GFP-DONSON distribution (enrichment = log2(ChIP/input)) in HeLa cells stably expressing GFP-DONSON. Some examples are shadowed in red of late-replicating regions aligning with FANCM enriched genomic regions. Some early-replicating regions are highlighted in blue showing correspondence with GFP-DONSON-enriched regions. In the RT profile, positive and negative values correspond to early and late replication, respectively. In the eigenvector profile, they correspond to the A and B compartments. Regions containing fragile sites are marked by red bars above the profiles. **b** Violin plot displaying the distribution of replication timing of 50-Kb genomic windows enriched in FANCM or GFP-DONSON ChIP (log2[ChIP/Input] > 0) and the A and B Hi-C compartments (eigenvector >0, <0, respectively), each compared with a matching number of randomly selected genomic windows of the same size. The box plot inside the violin plot shows the median (center line), the upper (Q3) and lower (Q1) quartiles (box bounds) and the highest and lowest values excluding outliers (extreme lines). **c** Coverage of H3K9me3, H3K4me3, FANCM, and GFP-DONSON of 50-Kb genomic windows within 25 replication-timing quantiles, going from late- to early-replicating regions, in HeLa cells expressing GFP-DONSON. **d** Coverage of H3K9me3, H3K4me3, FANCM, and GFP-DONSON of 50-kb genomic windows within 25 eigenvector quantiles, going from B to A Hi-C compartments in HeLa cells expressing GFP-DONSON. In the box plots in **c**, **d**, the center line represents the median, the box bounds represent the upper (Q3) and lower (Q1) quartiles, the extreme lines represent the highest and lowest value excluding outliers, and the black dot represents the mean.

The bias in replication timing and genome location indicated by the preceding experiments with untreated cells prompted a ChIP-seq analysis of DONSON and FANCM-associated DNA in cells without ICLs (see “Discussion”). Chromatin was prepared, sonicated, and immunoprecipitated against GFP-DONSON or FANCM. DNA was isolated and subjected to Next Gen sequence analysis. The enrichment of FANCM and GFP-DONSON [log2(ChIP/input)] across individual chromosomes was compared with the data on replication timing, and the Hi-C compartments A and B. The distribution of DONSON and FANCM-associated sequences in most regions in chromosomes such as 1, 5, 9 matched well with the replication timing and Hi-C A and B compartments, respectively (Fig. 6a; Supplementary Fig. 6a, c). Chromosomes, such as 6, 10, and 12, showed little overlap between DONSON and FANCM, but the correlations with early- and late-replicating DNA and the A and B compartments were not as strong (Supplementary Fig. 6b, d, e). In addition, there were chromosomes (14, 15) in which the DONSON and FANCM signals were intermingled (Supplementary Fig. 6f, g). There was no correspondence between the regions associated with DONSON or FANCM and fragile sites in any chromosome. Violin plots of DONSON and FANCM-associated DNA sequences (across the entire genome) that were enriched relative to the input were skewed toward sequences that were early replicating and in compartment A or late replicating and in compartment B, respectively (Fig. 6b).

In order to evaluate the relationship between DONSON and FANCM across the entire genome and early- or late-replicating loci, we calculated the coverage of the respective ChIP-seq results in replication-timing quantiles in the cells. As proof of principle, we also calculated the coverage of H3K9me3 and H3K4me3 histone marks, using the published data (Methods). As expected, the permissive chromatin mark H3K4me3 was progressively enriched toward early-replicating regions of the genome, while the repressive histone mark H3K9me3 was progressively enriched toward late-replicating regions. FANCM ChIP-seq data were increasingly enriched toward late-replicating regions, similar to H3K9me3 (Fig. 6c). The DONSON ChIP-seq showed a bias toward the quantiles that associated with early replication, although it was not as pronounced as the FANCM linkage to late replication. Another comparison was to the continuum of A–B chromatin compartments defined by Hi-C. Again, there was a clear bias in the sequences captured by FANCM toward the B compartment associated with silent chromatin and late-replicating sequences. DONSON-bound sequences were weighted toward the A compartment, but not as strongly as H3K4me3 (Fig. 6d).

## Discussion

In living cells, many more proteins associate with replisomes than are required for minimal biochemical reconstructions^9,33^. These interactions may be constitutive or induced by replication stress, but are typically interpreted as representing a single complex (see Introduction)^12,22,34–42^. An alternative view, that there are multiple, distinguishable, replisome variants, either constitutive or in response to stress, has received much less attention. Our results demonstrate two compositionally different replisomes in unstressed cells, and an additional two in cells containing potent blocks to replication. Furthermore, we find that the different replisomes are also distinguished by replication timing and chromatin location.

DONSON-bound replisomes were constitutively more prevalent in genomic regions with euchromatin histone marks, in chromatin compartment A, and were associated with early-replicating elements. FANCM was more frequent in heterochromatin, in compartment B, and biased toward late-replicating regions. The clarity of the data supporting these conclusions was dependent on experiments, in which cells from well separated stages of the S phase were analyzed (the PLA experiments), or chromatin complexes containing DONSON were separated from those bound by FANCM (the sequential IP). However, for practical reasons the ChIP-seq analyses were with unsorted cells which necessarily included cells from all stages of the S phase, blurring the distinction between early and late stages (see Supplementary Fig. 3e). Nonetheless, the ChIP-seq data, summed over the entire genome, were in accord with the conclusions of the experiments with early- and late-replicating cells. The examination of the patterns from individual chromosomes revealed some in excellent agreement with the early/late bias of DONSON/FANCM, while the results with others were not as clear. Generally, the distinctions were stronger for the FANCM-bound sequences than for those of DONSON, in agreement with the results from the PLA experiments. It should be noted that at best, these measurements will reflect the location of DONSON and FANCM in regions of the genome rather than at specific sites defined by several nucleotides, as would be the case with transcription factors. Factors that are involved in DNA transactions that function throughout the entire genome, or in enormous domains such as those assigned to the A and B compartments, are unlikely to be present at the same location at the same time across a cell population. These considerations have also been noted in analyses of the relationship between chromatin folding compartments and replication timing^12,35^.

The presence, in active chromatin, of constitutive DONSON replisomes (replisome: CMG-D) suggests a cellular anticipation of replication stress in regions that are more susceptible to DNA damage and collisions with transcriptional R loops^36–38^. Thus defects in DONSON^24,25^ would preferentially influence the response to replication stress in active gene regions of the genome. DONSON is mutant in a microcephalic dwarfism syndrome. Inefficiencies in transit through transcriptionally active gene regions^39^ could have adverse effects on completion of the S phase, and consequently cell number, resulting in the smaller brain and body size that are features of individuals with DONSON mutations.

On the other hand, our results indicate that in unstressed cells the association of FANCM with replisomes is infrequent. It is possible that the FANCM: replisomes in untreated cells result from encounters of replisomes with endogenous blocks. Our data demonstrate the bias of FANCM to regions that replicate late and are marked by histone modifications consistent with heterochromatin. Consequently, we suggest that replisomes that encounter blocks in these domains are in environments with associated FANCM, and ready targets for FANCM recruitment. These regions contain difficult to replicate sequences^31^. FANCM, which is an ancient protein with equivalents in archaea^21^, may have evolved, in part, to respond to replication blocks in sequences with a propensity to stall replication. In FANCM-deficiency disorders^40,41^, we would anticipate that the fault in at least a component of the response to replication stress would be in heterochromatin^42^.

In previous work, we showed that the FANCM increment of ICL traverse was dependent on the translocase activity, while the loss of GINS required only the association of the protein, including a translocase inactive version, with the replisome complex^23^. Thus, the functions of the protein in the traverse assay could be separated into at least two steps. We suggest that the replication restart pathway is multistep, requiring an opening of a gate in the replisome to allow the replisome to move past the ICL. Whether this is the gate between MCM2 and MCM5, which would be unlocked by the loss of the GINS^43^, or an alternative gate which can open independently of the GINS status^44,45^ remains to be determined^46^. In addition, the translocase activity of FANCM could be required for moving the opened replisome past the ICL or modulating DNA structure once past the barrier. These are not exclusive possibilities.

In contrast to FANCM, DONSON has no enzymatic activity. Consequently, it may serve as a recruitment platform for factors that promote the stability of replication forks that encounter obstacles in early and mid S phase. For example, these might include enzymes such as SMARCAL1 and ZRANB3 that protect forks from collapse, and would provide a translocase activity, perhaps similar to FANCM^47,48^. DONSON has also been demonstrated to be required for efficient activation of the ATR-dependent replication stress response^24^. Recent work suggests that it may have functions other than those specific to an active replisome^49,50^.

Euchromatin and heterochromatin domains are not absolute, but subject to alteration during development, neoplasia, and aging^31,51,52^. It will be of interest to determine the influence of these changes on the response to replication stress by DONSON and FANCM-associated replisomes.

## Methods

### Materials

Dig-TMP was synthesized by conjugation of an amine polyglycol with a chloro-TMP. The amine side chain was then reacted with a succinylester of digoxigenin to produce digoxigenin-TriMethyl Psoralen^53^. The siRNA for DONSON and FANCM were purchased from Dharmacon. L-017453-02-0005, ON-TARGETplus Human DONSON siRNA SMART pool (GAAAUCAUCUUUACGGAAU, UGGACAAAGUACUUGA UAU, GAGAUGGGUGUGCAAGAUA, ACUUAGUCAAAUACCGUUA). L-021955-00-0005, ON-TARGETplus Human FANCM siRNA—SMART pool (GGGUA GAACUGGCCGUAAA, GAGAGGAACGUAUUUAUAA, AAACAGACAUCGCUGAAUU, GCAUGUAGCUAGG AAGUUU). Other reagents were lipofectamine RNAiMAX (Invitrogen, 13778-150), Halt™ protease and phosphatase inhibitor cocktail (Thermo Scientific, 78446) and ATR inhibitor (VE821, Selleckchem, S8007).

### Antibodies

Rabbit oligoclonal anti-Dig, Invitrogen, Cat # 710019, dilution 1 in 200; rabbit polyclonal anti-FANCM, Bethyl, Cat # A302-637A, 1 in 1000; rabbit polyclonal to histone H3 (trimethyl K9), Abcam, Cat # ab8898, 1 in 500; rabbit polyclonal to histone H3 (trimethyl K4) antibody, Abcam, Cat # ab8580, 1 in 500; rabbit anti-MCM2, Abcam, Cat # ab4461, 1 in 200 for PLA, 1 in 1000; rabbit anti-MCM5, Abcam, Cat # ab17967, 1 in 200 for PLA, 1 in 1000; rabbit anti-MCM8, Proteintech, Cat # 16451-1-AP, 1 in 1000; rabbit anti-MCM10, Proteintech, Cat # 12251-1-AP, 1 in 1000; rabbit anti-phosphoMCM2S108, Abcam, Cat # ab109271, 1 in 200; rabbit anti-PSF1, Abcam, Cat # ab181112, 1 in 200 for PLA, 1 in 1000; rabbit anti-RAD51, Abcam, Cat # ab63801, 1 in 1000; mouse anti-FANCM, Sigma, Cat # SAB1407805, 1 in 100; rabbit anti-DONSON, from Andrew Jackson Laboratory, 1 in 500; mouse anti-GFP, Abcam, Cat # ab1218, 1 in 500; rabbit mAb anti-tubulin, Cell Signaling, Cat # 9099, 1 in 2000; rabbit mAb anti-GAPDH, Cell Signaling, Cat # 5174, 1 in 1000; rabbit anti-FANCM, Bethyl, Cat # 302-637A, 1 in 200; rabbit anti-DONSON, Sigma, Cat # HPA049033, 1 in 200; mouse anti-H3K4me3[304M3-B], absolute antibody, Cat # Ab00699-1.26, 1 in 500; mouse anti-H3K9me3[309M3-B], absolute antibody, Cat # Ab00700-1.26, 1 in 500. Duolink® in situ oligonucleotide PLA probe PLUS, Sigma-Aldrich, Cat # DUO92002, 8 µl per reaction; Duolink^®^ in situ oligonucleotide PLA probe MINUS, Sigma-Aldrich, Cat # DUO92004, 8 µl per reaction.

### Cells, cell culture, transfection

HeLa CCL-2 and RPE1 (ATCC) cells were maintained in DMEM (Gibco) supplemented with 10% fetal calf serum (Gibco), 100 U/mL penicillin, and 100 μg/mL streptomycin sulfate (Gibco). GFP-DONSON expressing HeLa cells^24^ were cultured with L-glutamine (Gibco), 200 μg/ml Hygromycin B (Invitrogen), and 5 μg/ml Blasticidin (Gibco). HeLa-Flp-In T-REx cells stably transfected with pcDNA5/FRT/TO-EGFP expressing EGFP or EGFP-DONSON were induced by incubation with 1 μg/ml doxycycline for 48 h. Cells derived from patient 9 with mutations in DONSON^24^, stably transduced with pMSCV-vector only or pMSCV-DONSON, were grown in DMEM (Gibco) supplemented with 10% fetal calf serum (Gibco), L-Glutamine (Gibco), 100 U/mL penicillin, 100 μg/mL streptomycin sulfate (Gibco). All cells were routinely tested for mycoplasma (Lonza, LT07-701). To determine the effect of knockdown of DONSON and FANCM in the DNA fiber assay, HeLa cells were transfected with 10 nM siRNA (Dharmacon) using RNAiMAX (Invitrogen) on days 1 and 2. Experiments were performed on day 4, ie.72 h after siRNA transfection.

### Chromatin extraction and immunoprecipitation

In total, 10^7^ cells were suspended in buffer A (10 mM HEPES at pH 7.9, 10 mM KCl, 1.5 mM MgCl2, 0.34 M sucrose, 10% glycerol, 1 mM DTT, 10 mM NaF, 1 mM sodium orthovanadate, 0.1% Triton X-100, with protease and phosphatase inhibitors), and incubated for 5 min on ice. Nuclei were recovered by centrifugation at 1300 *g* for 4 min. The nuclear pellet was lysed in buffer B (3 mM EDTA, 0.2 mM EGTA, 1 mM DTT, protease and phosphatase inhibitors) for 10 min on ice, and then centrifuged at 1700 *g* for 4 min. Chromatin was resuspended in benzonase buffer (Sigma, E8263, 250 U/mL benzonase, 20 mM Tris-HCl at pH 8.0, 0.2 mM MgCl_2_, 2 mM NaCl, protease and phosphatase inhibitors, and incubated at 4 °C overnight. Another 250 U/ml benzonase was added, and the sample was incubated for an additional 3 h. The sample was clarified by centrifugation, and the supernatant adjusted to 200 mM NaCl, 50 mM Tris-HCl pH 7.4, 0.1% Tween-20.

For immunoprecipitation, soluble chromatin samples were precleaned with Dynabeads Protein G (Life Technologies) for 1 h at room temperature, then incubated with specific antibodies at 4 °C overnight. For sequential Co-IP, immunoprecipitations were performed using protein G magnetic beads (Pierce, 10% v/v), GFP Trap (Chromotek, gta-20). We performed each immunocapture twice, in order to clear the target complex. After capture with one antibody was completed, the supernatant was incubated with the next antibody, and so on. All bead–antibody complexes were washed three times with PBST (phosphate buffered saline, .05% Tween-20. pH 7.5) and resuspended in SDS-PAGE loading buffer. After heating for 10 min at 90 °C, the proteins were analyzed by Western blotting according to standard procedures.

### In situ proximity ligation assay (PLA)

Cells were grown on Mattek glass bottomed plates followed by treatment with 5 µM Dig-TMP/UVA, 1.5 µM TMP/UVA, or UVA only. UVA exposure was in a Rayonet chamber at 3 J/cm^2^. After incubation with fresh medium for 60 min, cells were incubated with 0.1% formaldehyde for 5 min and then treated twice with CSK-R buffer (10 mM PIPES, pH 7.0, 100 mM NaCl, 300 mM sucrose, 3 mM MgCl_2_, 0.5% Triton X-100, 300 µg/ml RNAse), and fixed in 4% formaldehyde in PBS (W/V) for 10 min at RT, followed by incubation in pre-cold methanol for 20 min at −20 °C. After washing with PBS, the cells were treated with 100 ug/ml RNAse for 30 min at 37 °C. In situ PLA was performed using the Duolink PLA kit (Sigma-Aldrich) according to the manufacturer’s instructions. Briefly, the cells were blocked for 30 min at 37 °C and incubated with the respective primary antibodies (see reagent list) for 30 min at 37 °C. Following three times washing with PBST (phosphate buffered saline, 0.1% Tween), anti-mouse PLUS and anti-rabbit MINUS PLA probes were coupled to the primary antibodies for 1 h at 37 °C. After three times washing with buffer A (0.01 M Tris, 0.15 M NaCl, and 0.05% Tween-20) for 5 min, PLA probes were ligated for 30 min at 37 °C. After three times washing with buffer A, amplification using Duolink In Situ Detection Reagents (Sigma) was performed at 37 °C for 100 min. After amplification, the cells were washed for 5 min three times with wash buffer B (0.2 M Tris 0.1 M NaCl). Finally, they were coated with mounting medium containing DAPI (Prolong Gold, Invitrogen). Antibody specificity was confirmed by omitting one or another antibody. In some experiments, after completion of the PLA procedure, the stage of the S phase was determined by immunostaining of cells with an antibody against PCNA conjugated with Alexa 647.

### PLA imaging and quantification

PLA plates were imaged on a Nikon TE2000 spinning-disk confocal microscope, using a Plan Fluor ×60/1.25 numerical aperture oil objective. All images in an experiment were acquired with the same exposure parameters. Quantification was done on CellProfiler using the pipeline provided as Supplementary Software 1. Briefly, the pipeline performs the following steps: identify nuclei using the DAPI channel, filter to a maximum size the PLA foci, mask the foci image using the nuclei objects (PLA foci) to generate a visual representation of the foci counted for each cell, identify primary objects (PLA foci), establish a parent–child relationship between the foci (“children”) and nuclei (“parents”) in order to determine the number of foci per nucleus and export results as number of PLA foci per nucleus to a spreadsheet. The spreadsheets were compiled in Excel and exported to Graphpad Prism to generate the dot plots and determine if differences were statistically significant using the Mann–Whitney rank-sum test (NS: *P* > 0.5, significant: *P* < 0.001).

### Sequential PLA and 3D reconstruction

Mattek glass bottomed plates were marked on the growth surface with a diamond pen prior to plating cells in order to provide a reference for location of individual microscope fields^54^. GFP-DONSON: pMCM2S108 PLA was performed as above, with Duolink Detection Reagent Green (Sigma, DUO92014) or Orange (DUO92007). Bright-field images of the individual fields were obtained, as well as the patterns of the PLA. The plates were then incubated with 6 M guanidine: HCl in 5% of sucrose for 10 min at 40 πC to strip the antibodies and reaction products, and then washed with PBST. The fields were inspected to ensure complete removal of signal after which the FANCM: pMCM2S108 PLA was performed, with detection oligonucleotides linked to Duolink Detection Reagent Red (Sigma, DUO92013). The cells photographed after the first PLA were located and imaged again. In all, 16 stacks covering 1.6 μm were acquired of the first and second PLAs using Volocity software and exported as.OMETIFF. Both sets of images were converted into.ims to generate the 3D reconstructions on IMARIS (Bitplane) as follows. One of the sets (PLA2) was imported as a timepoint into the other set (PLA1). Next, a surface of each nuclei was created in the DAPI channel in order to track and correct for translational and rotational drift between the two PLA images. Each timepoint was then saved as the corresponding PLA, and the four channels were finally combined into one image to confirm correct alignment of nuclei (on the DAPI channel) and visualize the localization of both PLA signals on the same cell. A 3D reconstruction of a merged image is provided as Supplementary Movie 1.

### DNA fiber analysis

DNA fiber assays were performed as follows^19^: cells were incubated with 6 μM Dig-TMP at 37 °C for 1 h, followed by exposure to UVA light in a Rayonet chamber at 3 J/cm^2^ prior to incubation with 10 μM CldU for 20 min and then with 100 μM IdU for 20 min. Cells were trypsinized and suspended in PBS, and ~200 cells placed on a glass microscope slide (Newcomer Glass) and 10 μl of lysis buffer (0.5% SDS in 200 mM Tris-HCl pH 7.5, 50 mM EDTA) added. DNA fibers were spread and fixed in 3:1 methanol:acetic acid, denatured with 2.5 M HCl for 1 h, neutralized in 0.4 M Tris-HCl pH 7.5 for 5 min, washed in PBS, and immunostained using anti-Dig, anti-BrdU primary and corresponding secondary antibodies. Antibodies and dilutions were rat anti-BrdU (CldU), 1:200; Dylight 647 goat anti-rat, 1:100; mouse anti-BrdU (IdU), 1:40; and Dylight 488 goat anti-mouse, 1:100 and Q-dot 655 goat anti-mouse 1:2500. The slides were mounted in ProLong Gold Antifade Mounting medium. Images were acquired using a Zeiss Axiovert 200 M microscope at ×63 magnification with the Axio Vision software packages (Zeiss). The quantum dot signal was imaged with a Q-dot 655 filter.

### Analysis of early and late S-phase cells

GFP-DONSON expressing cells were treated with 1.5 μM TMP/UVA, and after 1 h were trypsinized and suspended in DMEM with 10% fetal calf serum and incubated with 16 µM Hoechst for 30 min at room temperature. The cells were centrifuged, washed with sorting buffer (HEPES pH 7.0, 1 mM EDTA, and 5% fetal calf serum), and then suspended in 1 ml of sorting buffer supplemented with 1 mM N-acetyl cysteine. The cells were then resolved by flow cytometry, and early and late S-phase fractions harvested. Gating was as follows: (1) Forward (FSC) and Side Scatter (SSC) to exclude debris, (2) FSC-A and FSC-H for doublet discrimination, (3) cells stained with Hoechst 33342, excited by ultraviolet light 355 nm, with an emission at 405 nm were gated based on DNA content into gate [R3] spanning late G1 and early S phase, and gate [R4], including cells spanning late S phase and G2. Gating example figures provided in Source Data for Fig. 3b.

Cells from each fraction were attached to slides by centrifugation (Cytospin), fixed with 0.1% formaldehyde and PLA between GFP-DONSON: pMCM2S108 or FANCM: pMCM2S108 performed. Cells were identified as early or late S phase according to their characteristic PCNA staining pattern.

### Chromatin immunoprecipitation (ChIP) for DNA analysis

Cells were crosslinked with 1% formaldehyde in culture media for 8 min, followed by quenching the formaldehyde with 0.1 M glycine. Cells were washed with PBS, harvested by scraping, then suspended in lysis buffer (0.5% SDS, 10 mM EDTA, 50 mM Tris-HCL pH 8.0) supplemented with protease and phosphatase inhibitors. Lysates were sonicated in a 4 πC water bath ultrasonicator (Bioruptor, Diagenode). The time of sonication was adjusted to yield short DNA fragments <500 bp (total 8 min, 30-s sonication, then cool 30 s). In some experiments, the time was adjusted to yield longer DNA fragments of 500–5000 bp (2 × 30 s with a 30-s cooling period). Diluted lysates were incubated overnight at 4 °C .with antibodies as indicated. Immunoprecipitations were performed using Protein G magnetic beads (Pierce, 10% v/v), or GFP Trap (Chromotek, gta-20). Bead-bound complexes were washed with low-salt immune-complex buffer (0.1% SDS, 1% Triton x-100, 2 mM EDTA, 20 mM Tris-HCl pH 8.0, 150 mM NaCl), high-salt immune-complex buffer (0.1% SDS, 1% Triton x-100, 2 mM EDTA, 20 mM Tris-HCl pH 8.0, 500 mM NaCl), LiCl immune-complex buffer (0.25 M LiCl, 1% NP-40, 1% mM EDTA, 10 mM Tris-HCl pH 8.0), and TE buffer (10 mM Tris-HCl, 1 mM EDTA pH 8.0). DNA was eluted in elution buffer (1% SDS, 0.2 M NaCl) with Proteinase K (100 µg/ml) overnight at 65 °C. Eluted DNA was purified with DNA Clean & Concentrator PCR purification Kit (ZYMO Research, D4033) according to the manufacturer instructions.

### Dot blot analysis

The DNA was denatured using 0.5 M NaOH and 1.5 M NaCl, and equal amounts were loaded onto a Hybond N + nitrocellulose membrane (GE Biosciences) using the Bio-Dot apparatus (Bio-Rad). Membranes were washed once with denaturing buffer and wash buffer (3× SSC), followed by UV-cross-linking (UV Stratalinker 1800, Stratagene) and blocking with 5× Denhardt’s solution (Thermo Scientific) for 1 h at 37 °C. Hybridization with Alu-Biotin (5′ biotin-GGCCGGGCGCGGTGGCTCACGCCTGTAATCCCAGCA), Satellite III (5′ biotin-TCCACTCGGGTTGATT) or LINE-1 (5′ biotin-GACTTCAAACTATACTACAAGGCTACA GTAACC) probes was performed at 37 °C overnight. Chemiluminescent Nucleic Acid Detection Module Kit (Thermo Scientific, 89880) was used for signal detection, and images were acquired using ChemiDox XRS with Image Lab software (Bio-Rad).

### Western blotting

For a full list of antibodies, see reporting summary. The samples were prepared in NuPAGE Sample Buffer (Invitrogen). Then proteins were separated by electrophoresis in 4–12% Bis-Tris Protein Gels and transferred to the polyvinylidene difluoride membrane (Thermo Scientific). The membranes were blocked in 5% dry milk in 0.1% Tween-20 in PBS and detected with the indicated antibodies. After incubation with horseradish peroxidase (HRP)-conjugated secondary antibodies (BIO-RAD), proteins were visualized using ECL detection reagents (GE Healthcare). Uncropped gel images for Western blots are available in Source Data file.

### Statistics and reproducibility

Statistical significance of PLA experiments was analyzed using the Mann–Whitney rank-sum test. Fiber patterns and immunoblotting were analyzed using a two-sided unpaired *t* test, and the exact *P*-values are given in each case. For both tests: significant: *P* < 0.001, NS (not significant): *P* > 0.05. All experiments were performed at least twice, and the number of biological replicates (*n*) is reported in each figure legend.

### ChIP-seq

Immunoprecipitation of sonicated chromatin was performed as described above.

### DNA sequencing

For DNA sequencing, Illumina sequencing adapters with a T-overhang were ligated to the precipitated ChIP DNA fragments or the input DNA, with a corresponding A-overhang, to construct a sequencing library according to the manufacturer’s protocol (Illumina, San Diego, CA). The fragments were purified using a magnetic bead protocol, and 18 cycles of PCR amplification were performed to enrich for fragments with an adapter on both ends. The products were purified again with size selection (~200–600 bases) using a dual-bead selection protocol with SPRIselect Beads (Beckman Coulter, Brea, CA). These libraries were sequenced on an Illumina Hi-Seq 2500 sequencer using on-board cluster generation on a rapid run paired end flow cell for 75 × 75 cycles (DONSON) and single end of 75 bp for 75 cycles (FANCM). Real-time analysis was performed using RTA v1.18.66.3, and base calling was performed using bcl2fastq v2.18.0.12.

### ChIP-seq, RT, and Hi-C data

The log2 ratio between FANCM or GFP_DONSON ChIP-seq and the Input was computed using Deeptools BigWigCompare of the corresponding RPKM normalized BigWig files. H3K9me3 and H3K4me3 ChIP-seq data from HeLa cells were downloaded from GEO: GSM2514495 and GSM3398459, respectively. Replication-timing data for HeLa S3 cells were downloaded from the replication-domain database, curated by the Gilbert laboratory (https://www2.replicationdomain.com/#: RT_HeLaS3_CervicalCarcinoma_Int 2355 8071_hg38). Hi-C data for HeLa cells were downloaded from NCBI, dbGaP phs000640.v8.p1 (10.1016/j.cell.2014.11.021). The eigenvector, used to delineate compartments in Hi-C data at coarse resolution, was calculated as the first principal component of the Pearson’s matrix using Juicer (-p KR, BP 50,000). UCSC liftover was used to convert hg19 to hg38 genome coordinates. Fragile sites mapping coordinates were downloaded from HumCFS: a database of Human chromosomal fragile sites (https://webs.iiitd.edu.in/raghava/humcfs/download.html).

### RT scores for FANCM and GFP-DONSON-enriched genomic windows

The genome was divided into 50-Kb windows, and the mean RT score for each window was calculated. The genomic regions enriched in FANCM and GFP-DONSON were selected as those 50-Kb windows in each ChIP with a [log2(ChIP/Input) > 0]. Their corresponding RT scores were determined, and their distribution of RT scores mapped as violin plots. The box plot inside the violins represent the median and the interquartile range. Randomly selected genomic regions, with number and size of the genomic windows matching each sample, were used as controls. All of the randomized samples have equivalent distributions. In the case of the eigenvector, we used 50-kb genomic regions with a value > 0 for the A compartment, and < 0 for the B compartment. As expected, the distribution of RT scores is heavily biased toward early replication for the A compartment, and late replication in the B compartment.

### ChIP-seq coverage of RT quantiles

The genome was divided into 50-Kb windows, and the mean RT score for each window was calculated. The coverage of each ChIP BAM file per genomic window was computed, and the counts converted to TPM (tags per million). The RT quantiles were calculated (*n* = 25). The ChIP-seq coverage was displayed in TPM for each of the 25 RT quantiles ordered from late to early.

### ChIP-seq coverage of Hi-C eigenvector quantiles

The eigenvector, used to delineate compartments in Hi-C data at coarse resolution, was calculated as the first principal component of the Pearson’s matrix using Juicer (-p KR, BP 50,000). ChIP-seq coverage of eigenvector quantiles was calculated as follows: the genome was divided into 50-Kb windows, and the mean eigenvector score for each window was calculated. We then computed the coverage of each ChIP-seq BAM file for each genomic window, converted counts to TPM (tags per million) and calculated Hi-C compartment eigenvector quantiles (*n* = 25). ChIP-seq coverage was displayed in TPM for each of the 25 eigenvector quantiles ordered from B to A.

### Data reporting

Statistical methods were not used for sample size determination. The experiments were not randomized, and the investigators were not blinded during experiments and data analysis.

### Data collection

Axiovisionx64 4.9.1.0, Zeiss, for acquisition of fiber images.

Volocity 6.5.1, Quorum Technologies for confocal microscopy image acquisition.

Summit V5.5.0.16880 software, for flow cytometry data.

Imaris ×64, 9.3.1, Bitplane, for correction of translational and rotational drift on sequential PLA images and 3D reconstruction.

CellProfiler 3.1.8, Opensource, for PLA quantification.

### Data analysis

Axiovisionx64 4.9.1.0, Zeiss, analysis of fiber images.

CellProfiler 3.1.8, Opensource, for PLA quantification.

Summit V5.5.0.16880 software for flow cytometry analysis.

Image Lab 5.1, gel densitometry analysis.

GraphPad Prism 7, numerical and statistical analysis.

Fastqc(0.11.9) for ChIP-seq data quality control.

BWA v. 0.7.17, ChIP-seq analysis.

SAMtools v.1.9, ChIP-seq analysis.

Picard v.2.9.2, ChIP-seq analysis.

BEDtools v.2.27.1, ChIP-seq analysis.

DeepTools v.3.0.1, ChIP-seq analysis.

Juicer v.1.5.6, eigenvector from Hi-C data.

R v.3.5.2, ChIP-seq analysis, RT quantile analysis and graphs.

UCSC toolkit v.388, ChIP-seq analysis.

Microsoft Excel for Microsoft 365 MSO.

CorelDraw X6, final figure assembly.

### Reporting summary

Further information on research design is available in the Nature Research Reporting Summary linked to this article.

## Supplementary information

Supplementary Information

Description of Additional Supplementary Files

Supplementary Movie 1

Supplementary Software 1

Reporting Summary

## Data Availability

All ChIP-seq and eigenvector datasets generated in this study are deposited in the NCBI Gene Expression Omnibus (GEO) database under accession number GSE150550. H3K9me3 and H3K4me3 ChIP-seq data from HeLa cells were downloaded from GEO database*:*GSM2514495 and GSM3398459, respectively. Replication-timing data for HeLa S3 cells were downloaded from the Replication Domain database, curated by the Gilbert laboratory (https://www2.replicationdomain.com/#: RT_HeLaS3_CervicalCarcinoma_Int 2355 8071_hg38). Hi-C data for HeLa cells were downloaded from NCBI, dbGaP phs000640.v8.p1. Fragile sites mapping coordinates were downloaded from HumCFS: a database of Human chromosomal fragile sites (https://webs.iiitd.edu.in/raghava/humcfs/download.html). The source data underlying Figs. 1b–f, 2b–c, 3b–f, 4a–d, 5a–d, and Supplementary Figs. 1c–g, 2a–f, 3c–e, 4a–c, and 5a,b are provided as a Source Data file. All data are available from the authors upon reasonable request.

## Source data

Source Data

## Code availability

The code used for ChIP-seq analysis has been deposited in Zenodo (doi: 10.5281/zenodo.3875101).
