# Supplementary material for: DONSON and FANCM associate with different replisomes distinguished by replication timing and chromatin domain
Source: Nat Commun. 2020 Aug 7;11:3951. doi: 10.1038/s41467-020-17449-1 (PMC7414851; doi:10.1038/s41467-020-17449-1)
Supplement: Supplementary file 1 — Supplementary Information [file 41467_2020_17449_MOESM1_ESM.pdf]

Supplementary Information for

DONSON and FANCM associate with different replisomes distinguished by replication timing and chromatin domain

by Zhang J. et al.

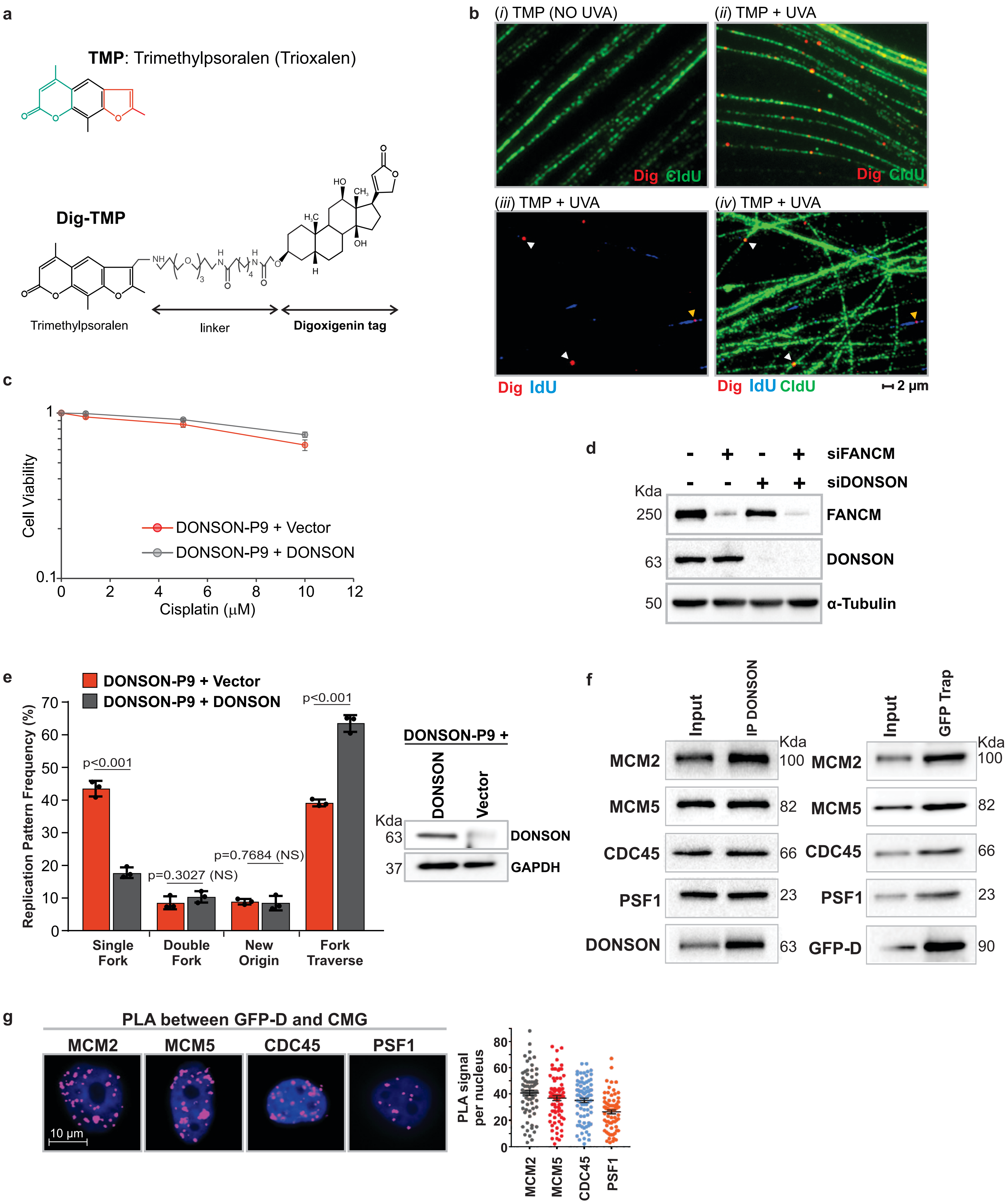

Supplementary Figure 1: DONSON contributes to replication traverse of ICLs. a. Structure of Digoxigenin-tagged trimethyl psoralen. **b**. Fibers from cells exposed to Dig-TMP/UVA, from 3 independent replicates. **i**. Cells were incubated with CldU 24 hrs,

then with Dig-TMP. The UVA exposure, required for crosslinking, was omitted. Fibers were displayed by immunofluorescence (green), and a primary antibody against Dig and a secondary tagged with Q-dot 655 (red). The absence of Q-dot signals reflects the absence of covalently bound Dig tagged ICLs. **ii**. Cells were incubated with 20 μM Dig/TMP (a higher concentration than in replication experiments) and exposed to UVA. Note the presence of numerous ICLs on the fibers. **iii**. Cells were incubated with CldU for 24 hrs, treated with 6 μM Dig-TMP/UVA, then incubated with IdU for 30 minutes. The IdU and Dig signals are shown, and an encounter with an ICL denoted (yellow arrow). Dig signals (white arrows) not associated with an IdU tract. **iv.** Display of CldU and IdU and Dig in the field shown in iii. Dig-TMP signals are on fibers labeled by CldU, although they may not be associated with an IdU tract (yellow arrow). **c**. DONSON does not contribute to survival of cells exposed to Cisplatin. P9 Patient derived cells, with severely reduced levels of DONSON protein, were complemented with either wild type DONSON or vector only. Statistical analysis was performed using unpaired, two-sided Student's t-test. Data are mean ± s.d, from 3 independent replicates. **d**. Knockdown efficiency of siRNA against FANCM, DONSON, or FANCM/DONSON in HeLa cells. Representative blot (n = 3). **e**. Single fork stalling at ICLs is increased in P9 patient derived cells. The replication traverse assay was performed in patient derived cells complemented with the vector alone or wild type DONSON. Quantitation of pattern distribution from cells treated as indicated. Fibers with ICL encounters: DONSON-P9-vector= 397, DONSON-P9-DONSON= 416, from 3 independent replicates. Data are mean ± s.d. A two-sided unpaired t-test was used to determine if differences were statistically significant. NS, not significant: p>0.05. Representative blot (n = 3). **f**. Immunoprecipitation of either endogenous DONSON or GFP-DONSON demonstrates association with replisome proteins. Representative blot (n = 3). **g**. The proximity of GFP-DONSON and replisome proteins demonstrated by PLA. Scored nuclei: GFP-DONSON and MCM2= 76, GFP-DONSON and MCM5= 79, GFP-DONSON and CDC45= 80, GFP-DONSON and PSF1= 76, from 3 biological replicates. Source data are provided as a Source Data file.

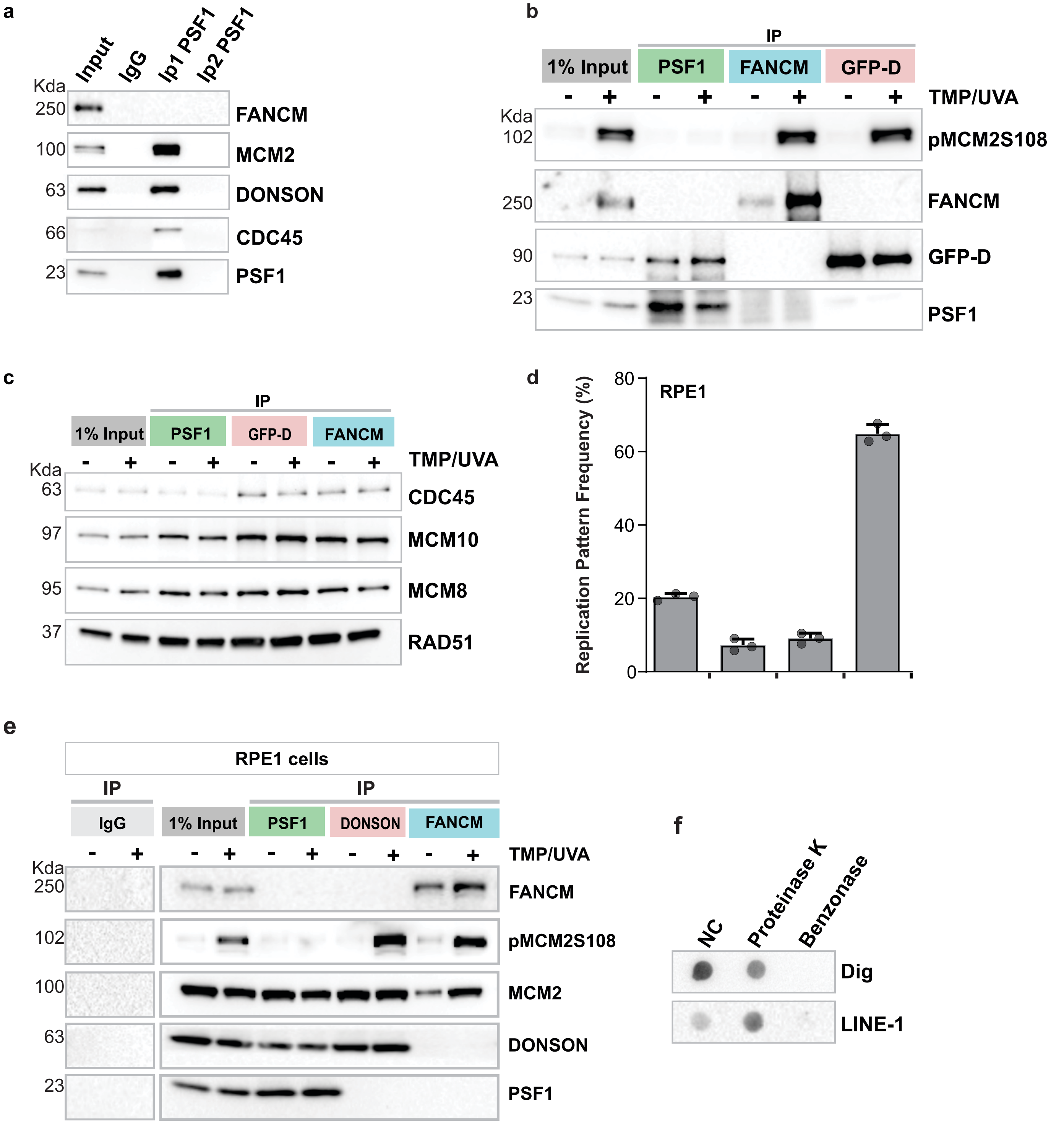

Supplementary Figure 2: DONSON and FANCM are on different replisomes. a. Efficacy of IP against the GINS protein PSF1. Chromatin proteins were incubated with antibody against PSF1 and the precipitate removed. No PSF1 was recovered when the supernatant was challenged again with the same antibody. Representative blot (n = 2). **b.** Reversal of the order of the sequential IP of replisome components does not change the results. IP against FANCM preceded IP against GFP-DONSON. Representative blot (n = 2). **c.** Proteins common to each replisome complex. Representative blot (n = 3). **d.** Replication patterns in RPE1 cells containing ICLs are the same as in other cells. Replication patterns in RPE1 cells containing ICLs are the same as in other cells. Fibers with ICL encounters n=475, from 3 independent replicates. Data are mean ± s.d. A two-sided unpaired t-test was used to determine if differences were statistically significant. NS, not significant: p>0.05. **e.** DONSON and FANCM are on different replisomes in RPE1 cells. Representative blot (n = 3). **f.** Dig-TMP in sonicated chromatin is associated with DNA. To verify the covalent linkage of Dig-ICL with DNA the sonicated chromatin used for sequential IP was digested with either benzonase or proteinase K. The samples were then examined by dot blot for the Dig tag on the ICLs. Representative blot (n = 3). Source data are provided as a Source Data file.

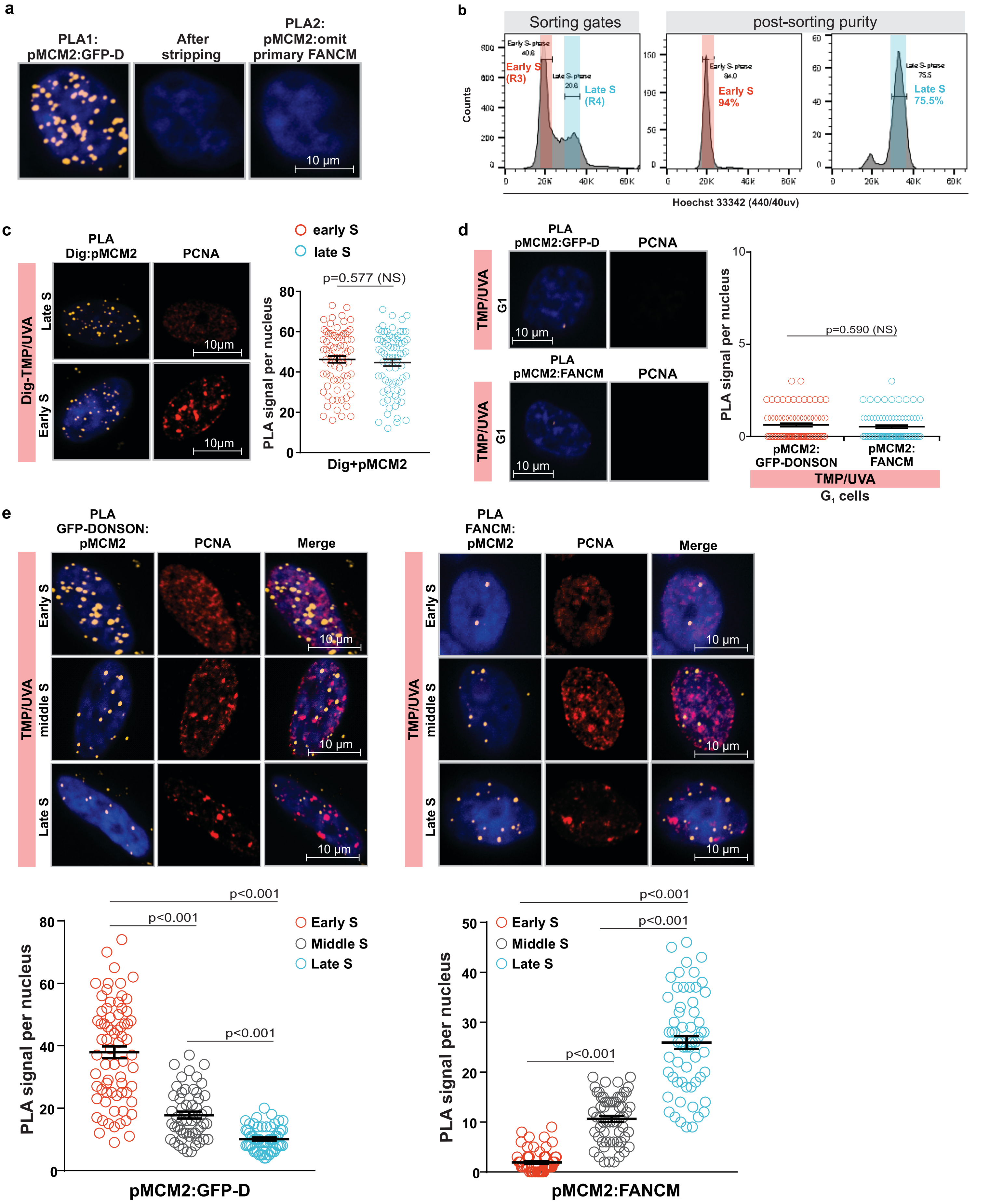

Supplementary Figure 3: DONSON and FANCM replisomes are in different locations and active at different times of S phase. a. Antibody omission control for sequential PLA. Accurate interpretation of sequential PLA (Methods) requires complete

removal of the antibodies and reaction products of the first reaction, in order to avoid compromising the second reaction. The GFP-DONSON: pMCM2S108 PLA was performed. After image acquisition the cells were stripped of the components and products of the PLA and a second PLA was performed without the addition of the antibody against FANCM. The absence of signal demonstrates the efficacy of the stripping procedure from 3 independent experiments. **b**. Sorted early and late S phase cells are not cross contaminated. Early and late S phase cells were recovered and re-analyzed. Contour plots showing the gating strategy can be found in the Source Data file. **c**. The frequency of replisome encounters with ICLs is similar in early and late S phase. The PLA between pMCM2 and the Dig tag on the ICLs shows equivalent frequencies in early and late S phase. Scored nuclei of PLA between pMCM2S108 and Dig tag in early S phase= 76, late S phase= 75, from 3 biological replicates. Data are mean ± s.e.m. **d**. Specificity test of DONSON and FANCM PLA with pMCM2S108. There can be no replisome encounters with ICLs in G1 phase cells. The PLA between GFP-DONSON or FANCM and pMCM2S108 was performed as a test of antibody and assay specificity. Scored nuclei of PLA between GFP-D and pMCM2S108= 75, FANCM and pMCM2S108= 75 from 3 biological replicates. Data are mean ± s.e.m. **e**. The distinction between DONSON: pMCM2 replisomes and FANCM: pMCM2 replisomes in early and late S phase is lost in mid S phase cells. Scored nuclei: GFP-D and pMCM2S108, early S phase= 70, middle S phase= 53, late S phase= 59; FANCM and pMCM2S108, early S phase= 64, middle S phase= 61, late S phase= 60, from 3 biological replicates. Data are mean ± s.e.m. For PLA experiments in (c-e) a two-sided Mann-Whitney Rank sum test was used to determine if differences were statistically significant. NS, not significant: $p>0.05$. Source data are provided as a Source Data file.

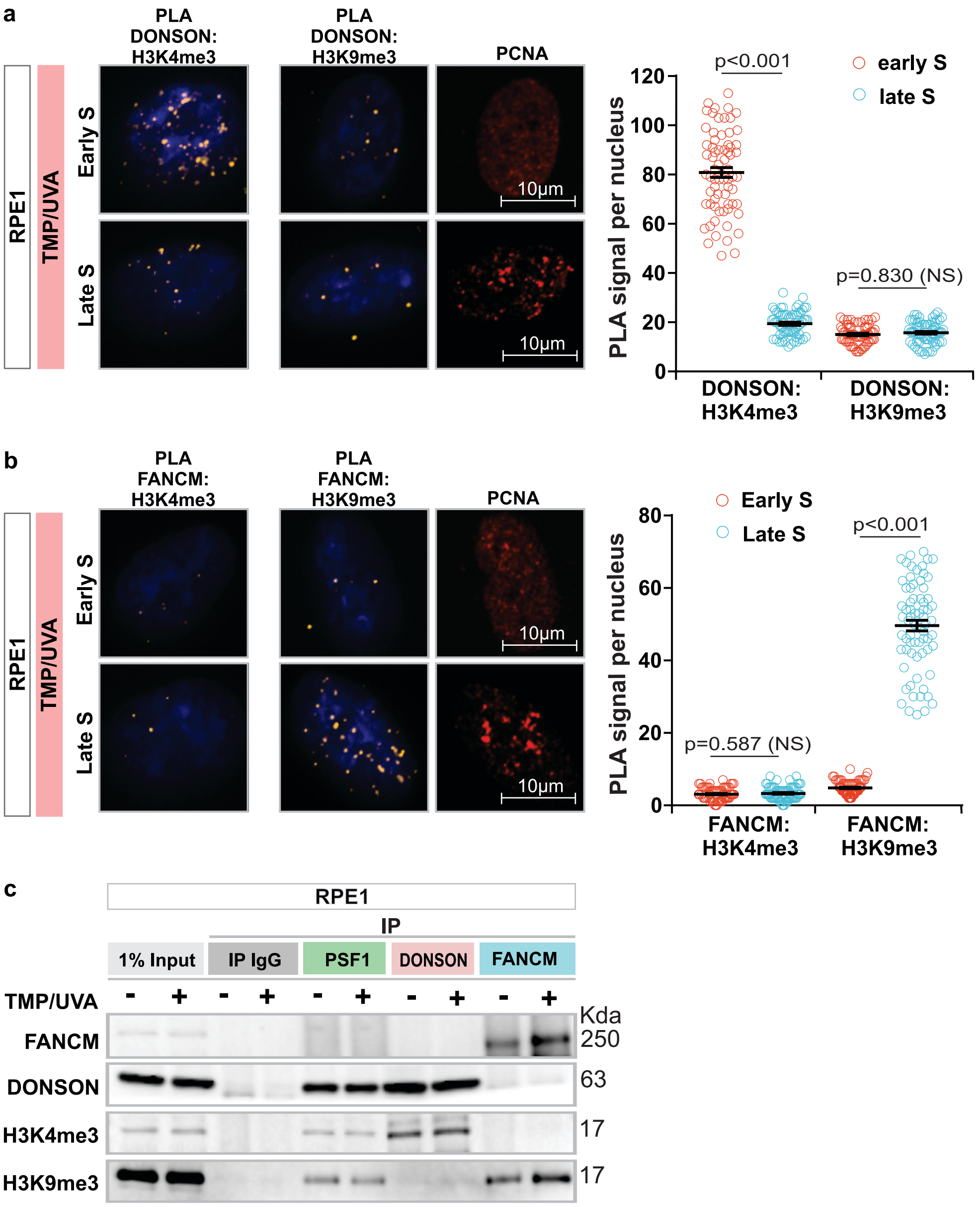

Supplementary Figure 4: Association of DONSON and FANCM with H3K4m3 and H3K9me3 in RPE1 cells. RPE1 cells were treated with TMP/UVA and PLA between endogenous DONSON or FANCM and H3K4me3 or H3K9me3 performed. Signals were quantitated in early or late S phase cells. **a.** The association of DONSON with H3K4me3 is greater in early S phase cells than in late S phase. The interaction of DONSON with H3K9me3 is low in both early and late S phase cells. Scored nuclei: DONSON and H3K4me3, early S phase= 62, late S phase= 64; DONSON and H3K9me3, early S phase= 63, late S phase= 60, from 3 biological replicates. Data are mean ± s.e.m. **b**. The association of FANCM with H3K4me3 is low in both early and late S phase, while that with H3K9me3 is much stronger in late than in early S phase. Scored nuclei: FANCM and H3K4me3, early S phase= 69, late S phase= 70; DONSON and H3K9me3, early S phase= 73, late S phase= 70, from 3 biological replicates. Data are mean ± s.e.m. **c**. Cells were exposed to UVA or TMP/UVA. Sequential IP reveals greater association of DONSON with H3K4me3 than H3K9me3, and greater association of FANCM with H3K9me3 than H3K4me3. Representative blot (n = 3). For PLA experiments in (a, b) a two-sided Mann-Whitney Rank sum test was used to determine if differences were statistically significant. NS, not significant: p>0.05. Source data are provided as a Source Data file.

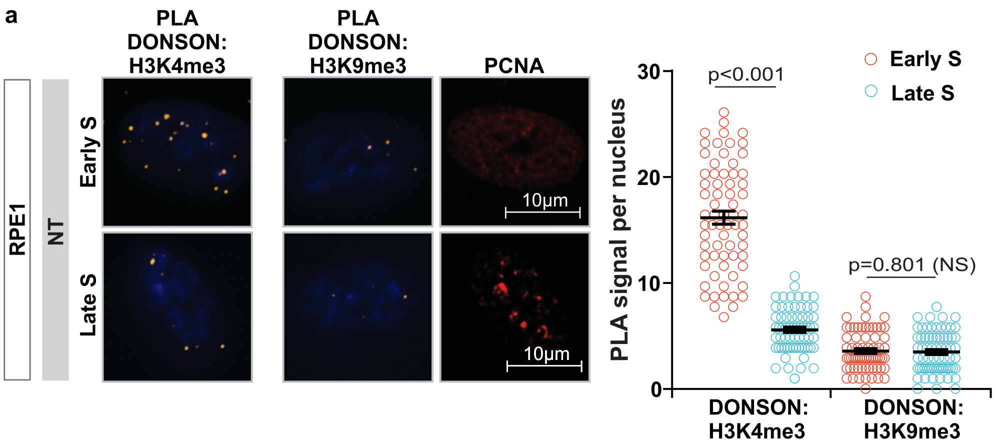

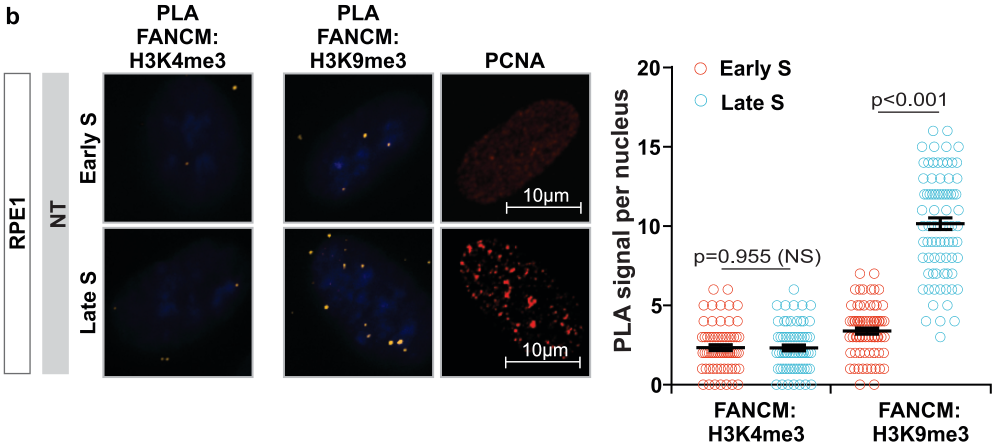

Supplementary Figure 5: Interactions between DONSON or FANCM and H3K4me3 or H3K9me3 in untreated early and late S phase RPE cells. a. PLA between endogenous DONSON and H3K4me3 or H3K9me3. The association of DONSON with H3K4me3 is greater in early S phase cells than in late S phase. The interaction of DONSON with H3K9me3 is low in both early and late S phase cells. Scored nuclei: DONSON and H3K4me3, early S phase= 68, late S phase= 65; DONSON and H3K9me3, early S phase= 64, late S phase= 68, from 3 biological replicates. Data are mean ± s.e.m. **b**. PLA between FANCM and H3K4me3 or H3K9me3. The association of FANCM with H3K4me3 is low in both early and late S phase, while that with H3K9me3 is much stronger in late than in early S phase. Scored nuclei: FANCM and H3K4me3, early S phase= 67, late S phase= 65; DONSON and H3K9me3, early S phase= 73, late S phase= 80, from 3 biological replicates. Data are mean ± s.e.m. For PLA experiments in (a-b) a two-sided Mann-Whitney Rank sum test was used to determine if differences were statistically significant. NS, not significant: $p>0.05$. Source data are provided as a Source Data file.

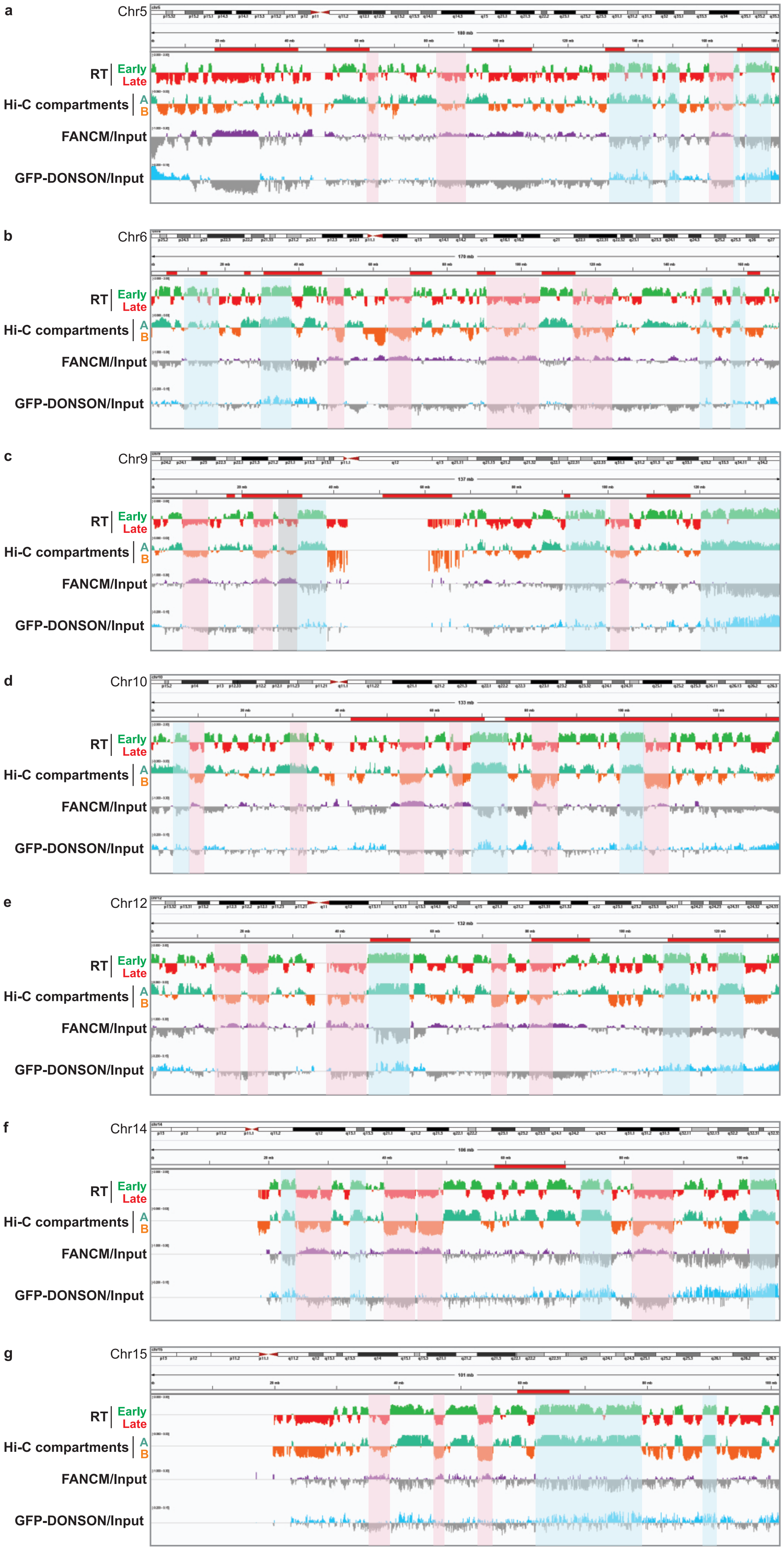

Supplementary Figure 6: CHIP-seq distribution profiles for selected chromosomes. Correlations between FANCM and late replicating regions and chromatin compartment B are indicated in pink. Correlations of DONSON with early replicating regions and chromatin compartment A are indicated in blue. **a.** Chr 5. **b.** Chr 6. **c.** Chr 9. **d.** Chr 10. **e.** Chr 12. **f.** Chr 14. **g.** Chr 15.
