# Supplementary material for: DONSON and FANCM associate with different replisomes distinguished by replication timing and chromatin domain
Source: Nat Commun. 2020 Aug 7;11:3951. doi: 10.1038/s41467-020-17449-1 (PMC7414851; doi:10.1038/s41467-020-17449-1)
Supplement: Supplementary file 2 — Description of Additional Supplementary Files [file 41467_2020_17449_MOESM2_ESM.pdf]

Description of Additional Supplementary Files

File Name: Supplementary Data 1

Description: **Video with 3D reconstruction of a cell showing sequential PLA signals do not overlap:** During the first 2 seconds 4 channels can be seen: PLA1 (pMCM2: DONSON PLA in red) with associated DAPI (blue) and PLA2 (pMCM2: FANCM PLA in green) with associated DAPI (light blue). One of the DAPI channels is then turned off and on to show the correct alignment of the nucleus captured in the sequential PLAs. At 0:7sec, the PLA2 signal is turned off and on, and then the nucleus is rotated 365 degrees horizontally to allow visualization of the lack of overlap between the two PLAs signals.

File Name: Supplementary Software 1

Description: **Cellprofiler pipeline for PLA quantification:** Briefly, the pipeline performs the following steps: identify nuclei using the DAPI channel, filter to a maximum size the PLA foci, mask the foci image using the nuclei objects (PLA foci) to generate a visual representation of the foci counted for each cell, identify primary objects (PLA foci), establish a parent-child relationship between the foci ("children") and nuclei ("parents") in order to determine the number of foci per nucleus and export results as number of PLA foci per nucleus to a spreadsheet.
