# Supplementary material for: DONSON and FANCM associate with different replisomes distinguished by replication timing and chromatin domain
Source: Nat Commun. 2020 Aug 7;11:3951. doi: 10.1038/s41467-020-17449-1 (PMC7414851; doi:10.1038/s41467-020-17449-1)
Supplement: Supplementary file 5 — Reporting Summary [file 41467_2020_17449_MOESM5_ESM.pdf]

nature research

Corresponding author(s): Michael Seidman

Last updated by author(s): June 4, 2020

Reporting Summary

Nature Research wishes to improve the reproducibility of the work that we publish. This form provides structure for consistency and transparency in reporting. For further information on Nature Research policies, see our Editorial Policies and the Editorial Policy Checklist.

Statistics

For all statistical analyses, confirm that the following items are present in the figure legend, table legend, main text, or Methods section.

n/a	Confirmed	
☐	☒	The exact sample size (*n*) for each experimental group/condition, given as a discrete number and unit of measurement
☐	☒	A statement on whether measurements were taken from distinct samples or whether the same sample was measured repeatedly
☐	☒	The statistical test(s) used AND whether they are one- or two-sided<br>*Only common tests should be described solely by name; describe more complex techniques in the Methods section.*
☒	☐	A description of all covariates tested
☒	☐	A description of any assumptions or corrections, such as tests of normality and adjustment for multiple comparisons
☐	☒	A full description of the statistical parameters including central tendency (e.g. means) or other basic estimates (e.g. regression coefficient) AND variation (e.g. standard deviation) or associated estimates of uncertainty (e.g. confidence intervals)
☐	☒	For null hypothesis testing, the test statistic (e.g. *F*, *t*, *r*) with confidence intervals, effect sizes, degrees of freedom and *P* value noted<br>*Give P values as exact values whenever suitable.*
☒	☐	For Bayesian analysis, information on the choice of priors and Markov chain Monte Carlo settings
☒	☐	For hierarchical and complex designs, identification of the appropriate level for tests and full reporting of outcomes
☒	☐	Estimates of effect sizes (e.g. Cohen's *d*, Pearson's *r*), indicating how they were calculated

Our web collection on statistics for biologists contains articles on many of the points above.

Software and code

Policy information about availability of computer code

Data collection	Axiovisionx64 4.9.1.0, Zeiss, for acquisition of fiber images<br>Volocity 6.5.1, Quorum Technologies for confocal microscopy image acquisition<br>Summit V5.5.0.16880 software, for flow cytometry data<br>Imaris ×64, 9.3.1, Bitplane, for correction of translational and rotational drift on sequential PLA images and 3D reconstruction<br>CellProfiler 3.1.8, Opensource, for PLA quantification

Data analysis	Axiovisionx64 4.9.1.0, Zeiss, analysis of fiber images<br>CellProfiler 3.1.8, Opensource, for PLA quantification<br>Summit V5.5.0.16880 software for flow cytometry analysis<br>Image Lab 5.1, gel densitometry analysis<br>GraphPad Prism 7, numerical and statistical analysis<br>Fastqc(0.11.9) for CHIp-seq data quality control<br>BWA v. 0.7.17, CHIP-seq analysis<br>SAMtools v.1.9, CHIP-seq analysis<br>Picard v.2.9.2, CHIP-seq analysis<br>BEDtools v.2.27.1, CHIP-seq analysis<br>DeepTools v.3.0.1, CHIP-seq analysis<br>Juicer v.1.5.6, eigen vector from Hi-C data<br>R v.3.5.2, CHIP-seq analysis, RT quantile analysis and graphs<br>UCSC toolkit v.388, CHIP-seq analysis<br>Microsoft Excel for Microsoft 365 MSO<br>CorelDraw X6, final figure assembly

For manuscripts utilizing custom algorithms or software that are central to the research but not yet described in published literature, software must be made available to editors and reviewers. We strongly encourage code deposition in a community repository (e.g. GitHub). See the Nature Research guidelines for submitting code & software for further information.

Data

Policy information about availability of data

All manuscripts must include a data availability statement. This statement should provide the following information, where applicable:

- Accession codes, unique identifiers, or web links for publicly available datasets
- A list of figures that have associated raw data
- A description of any restrictions on data availability

All CHIp-seq and eigenvector datasets generated in this study are deposited in the NCBI Gene Expression Omnibus (GEO) database under accession number GSE150550.

Field-specific reporting

Please select the one below that is the best fit for your research. If you are not sure, read the appropriate sections before making your selection.

☒ Life sciences ☐ Behavioural & social sciences ☐ Ecological, evolutionary & environmental sciences

For a reference copy of the document with all sections, see nature.com/documents/nr-reporting-summary-flat.pdf

Life sciences study design

All studies must disclose on these points even when the disclosure is negative.

Sample size	Sample size was not predetermined by statistical method as this work did not involve animal models or human subjects. The number of cells (PLA, n = 55-426) or replication patterns (DNA fibers, n = 380-430) per experiment were chosen based on established best practices in the field (Sodeberg, O. et al. 2006, Nature Methods 3, 995-1000; Huang, J. et al. 2013, Mol. Cell 52(3): 434-46; Mutreja, K. et al. 2018, Cell Rep. 24(10): 2629-2642).
Data exclusions	No data were excluded from the analysis
Replication	All DNA fiber, PLA and chromatin immunoprecipitation experiments were independently replicated on different days and at least two independent experiments. The number of biological replicates (n) is reported in each figure legend. All attempts at replication were successful.
Randomization	No human participants or animal models were reported in this manuscript. Randomization was not necessary. Experiments supporting main conclusions were replicated in two different cell lines. Appropriate controls were included for each experiment, such as UVA only treatment (PLA, Replication patterns), normal IgG control (chromatin IP, WB), cells transfected with Non-Targeting control (NT) siRNA (Replication patterns), Input DNA (ChIp-seq coverage of RT and Hi-C eigen vector quantiles).
Blinding	Formal blinding was not done but, whenever possible, experiments were repeated by different researchers. In all cases results were consistent between researchers.

Reporting for specific materials, systems and methods

We require information from authors about some types of materials, experimental systems and methods used in many studies. Here, indicate whether each material, system or method listed is relevant to your study. If you are not sure if a list item applies to your research, read the appropriate section before selecting a response.

Materials & experimental systems

n/a	Involved in the study
☐	☒ Antibodies
☐	☒ Eukaryotic cell lines
☒	☐ Palaeontology and archaeology
☒	☐ Animals and other organisms
☒	☐ Human research participants
☒	☐ Clinical data
☒	☐ Dual use research of concern

Methods

n/a	Involved in the study
☐	☒ ChIP-seq
☐	☒ Flow cytometry
☒	☐ MRI-based neuroimaging

Antibodies

Antibodies used

Rat mAb anti-BrdU (detecting CldU), Abcam, Cat# ab6326, 1 in 200, RRID: AB_305426
mouse anti-BrdU (detecting IdU), BD Biosciences, 1 in 40, Cat# 347580, RRID: AB_400326
Rabbit mAb anti CDC45, Abcam, Cat# ab126762, 1 in 200 for PLA, 1 in 1000 for WB, RRID: AB_11140216
Rabbit oligoclonal anti Digoxigenin (Dig), Invitrogen, Cat# 710019, 1 in 200, RRID: AB_2532530
Rabbit polyclonal anti FANCM, Bethyl, Cat# A302-637A, 1 in 1000, RRID: AB_10567252
Rabbit polyclonal to Histone H3 (tri methyl K9), Abcam, Cat# ab8898, 1 in 500, RRID: AB_306848
Rabbit polyclonal to Histone H3 (tri methyl K4) antibody, Abcam, Cat# ab8580, 1 in 500, RRID: AB_306649
Rabbit anti MCM2, Abcam, Cat# ab4461,1 in 200 for PLA, 1 in 1000 for WB, RRID: AB_304470
Rabbit anti MCM5, Abcam, Cat# ab17967, 1 in 200 for PLA, 1 in 1000 for WB, RRID: AB_444144
Rabbit anti MCM8, Proteintech, Cat# 16451-1-AP, 1 in 1000, RRID: AB_2142833
Rabbit anti MCM10, Proteintech, Cat# 12251-1-AP, 1 in 1000, RRID: AB_2266259
AF647-Rabbit anti PCNA, Cat# sc-56, Santa Cruz, sc-56 AF647, 1 in 200, RRID: AB_628110
Rabbit anti phosphoMCM2S108, Abcam, Cat# ab109271, 1 in 200 for PLA, 1 in 1000 for WB, RRID: AB_10891758
Rabbit anti PSF1, Abcam, Cat# ab181112, 1 in 200 for PLA, 1 in 1000 for WB, RRID N/A
Rabbit anti RAD51, Abcam, Cat# ab63801, 1 in 1000, RRID: A B_1142428
Mouse anti FANCM, Sigma, Cat# SAB1407805, 1 in 100, RRID: AB_10760703
Rabbit anti DONSON, from Andrew Jackson laboratory, 1 in 500, N/A
Mouse anti GFP, Abcam, Cat# ab1218, 1 in 500 for PLA, 1 in 2000 for WB, RRID: AB_298911
Rabbit mAb anti -Tubulin, Cell Signaling, Cat# 9099, 1 in 2000, RRID: AB_10695471
Rabbit mAb anti GAPDH, Cell Signaling, Cat#5174, 1 in 1000, RRID: AB_10622025
Mouse IgG Control, Abcam, Cat# ab18413, 1 in 50, RRID: AB_2631983
VeriBlot for IP Detection Reagent (HRP), Abcam, Cat# ab131366, 1 in 200, N/A
AF488-Goat anti mouse IgG, ThermoFisher, Cat# A-11001, 1 in 1000, RRID: AB_2534069
AF647-Goat anti Rat IgG, ThermoFisher, Catalog # A-21247, 1 in 1000, RRID: AB_141778
Qdot655-Goat anti rabbit IgG, ThermoFisher, Cat# Q-11421MP, 1 in 2000, RRID: AB_1500767
AF633-Goat anti Rat IgG, ThermoFisher, Cat# A-21094, 1 in 1000, RRID: AB_2535749
Rabbit anti-FANCM, Bethyl, Cat#302-637A, 1 in 200, RRID: AB_10567252
Rabbit anti-DONSON, Sigma, Cat#HPA049033, 1 in 200, RRID: AB_2680608
Mouse anti-H3K4me3[304M3-B], Absolute, Cat#Ab00699-1.26, 1 in 500.
Mouse anti-H3K9me3[309M3-B], Absolute, Cat#Ab00700-1.26, 1 in 500.
Duolink® in situ oligonucleotide PLA probe PLUS, Sigma-Aldrich, Cat#DUO92002, 8 µl per reaction, RRID: AB_2810940
Duolink® in situ oligonucleotide PLA probe MINUS, Sigma-Aldrich, Cat#DUO92004, 8 µl per reaction, RRID: AB_2713942

Validation

Validation acronyms: Immunoprecipitation (IP), immunofluorescence (IF), Western-blot (WB), ImmunoHistoChemistry (IHC), Immunoytochemistry (ICC).

-Rat anti-BrdU antibody, Validated by the company and cited in 724 publications. Statement from Abcam, validated for ICC/IF, IHC, Flow Cyt, ELISA, IHC-FrFl, https://www.abcam.com/brdu-antibody-bu175-icr1-proliferation-marker-ab6326.html
-Mouse anti-BrdU antibody, Monoclonal antibody firstly described in Science 1982, and cited in 7 publications. Statement from BD, validated for IF. https://www.bdbiosciences.com/us/applications/research/apoptosis/purified-antibodies/purified-mouse-anti-brdu-b44/p/347580
-Rabbit mAb anti CDC45, Validated by the company, cited in Molecular Cell 52(3):434-46. Statement from Abcam, validated for ICC/IF, IHC-P, Flow Cyt, WB. https://www.abcam.com/cdc45l-antibody-epr5759-ab126762.html
-Rabbit oligoclonal anti Digoxigenin (Dig), Validated by the company and cited in Molecular Cell 52(3):434-46. Statement from Thermofisher, validated for WB. https://www.thermofisher.com/antibody/product/Digoxigenin-Antibody-clone-9HCLC-Recombinant-Polyclonal/710019
-Rabbit polyclonal anti FANCM, Validated by the company. Statement from BETHYL, validated for IP, WB. https://www.bethyl.com/product/A302-637A/FANCM+Antibody
-Rabbit polyclonal to Histone H3 (tri methyl K9), Validated by the company and cited in 1015 publications. Statement from Abcam, validated for ChIP, ChIP/Chip, CHIPseq, Flow Cyt, ICC/IF, IHC-Fr, IHC-P, WB. https://www.abcam.com/products?keywords=Cat%23+ab8898
-Anti-Histone H3 (tri methyl K4) antibody, Validated by the company and cited in 1063 publications. Statement from Abcam, validated for ChIP, ChIP/Chip, CHIPseq, Flow Cyt, ICC, ICC/IF, IHC-Fr, IHC-P, IP, PepArr, WB. https://www.abcam.com/products?keywords=Cat%23+ab8580
-Rabbit anti MCM2, Validated by the company and cited in 23 publications. Statement from Abcam, validated for IHC-Fr, ICC/IF, IHC-P, WB, IP. https://www.abcam.com/mcm2-antibody-ab4461.html
-Rabbit anti MCM5, Validated by the company and cited in 15 publications. Statement from Abcam, validated for WB, IP, ICC/IF, IHC-

P. https://www.abcam.com/mcm5-antibody-ab17967.html
-Rabbit anti MCM8, Validated by the company. Statement from Proteintech, validated for WB, IP. https://www.ptglab.com/products/MCM8-Antibody-16451-1-AP.html
-Rabbit anti MCM10, Validated by the company and cited in 6 publications. Statement from Proteintech, validated for WB, IF. https://www.ptglab.com/Products/MCM10-Antibody-12251-1-AP.html
-AF647-Rabbit anti PCNA, Validated by the company and cited in 1911 publications. Statement from Santa Cruz, validated for IHC-Fr, IHC-P, IP, WB. https://www.scbt.com/p/pcna-antibody-pc10
-Rabbit anti phosphoMCM2S108, Validated by the company and cited in Molecular Cell 52(3):434-46. Statement from Abcam, validated for WB, IP, ICC/IF, IHC-P. https://www.abcam.com/mcm2-phospho-s108-antibody-epr4121-ab109271.html
-Rabbit anti PSF1, Validated by the company and cited in Molecular Cell 52(3):434-46. Statement from Abcam, validated for Flow Cyt, WB, ICC/IF. https://www.abcam.com/psf1-antibody-epr13359-ab181112.html
-Rabbit anti RAD51, Validated by the company and cited in 6 publications. Statement from Abcam, validated for IP, WB, ICC/IF. https://www.abcam.com/rad51-antibody-ab63801.html
-Mouse anti FANCM, Validated by the company. Statement from Sigma, validated for IP, WB, IF. https://www.sigmaaldrich.com/catalog/product/sigma/sab1407805?lang=en&region=US
-Rabbit anti DONSON, Validated and cited in Nature Genetics, 49:537-549. -Mouse anti GFP, Validated by the company and cited in 236 publications. Statement from Abcam, validated for WB, CHIP, Sandwich ELISA, ICC/IF, IP. https://www.abcam.com/gfp-antibody-9f9f9-ab1218.html
-Rabbit mAb anti -Tubulin, Validated by the company and cited in 22 publications. Statement from Cell Signaling, validated for WB, IHC-Fr, ELISA, ICC/IF, IP. https://www.cellsignal.com/products/antibody-conjugates/a-tubulin-11h10-rabbit-mab-hrp-conjugate/9099
-Rabbit mAb anti GAPDH, Validated by the company and cited in 1213 publications. Statement from Cell Signaling, validated for WB, IHC, IF. https://www.cellsignal.com/products/primary-antibodies/gapdh-d16h11-xp-rabbit-mab/5174?site-search-type=Products
-Mouse IgG Control, Validated by the company and cited in 22 publications. Statement from Abcam, validated for Flow Cyt, ChIP/Chip, ChIP. https://www.abcam.com/mouse-igg2a-kappa-monoclonal-mopc-173-isotype-control-chip-grade-ab18413.html
-VeriBlot for IP Detection Reagent (HRP), Validated by the company and cited in 28 publications. Statement from Abcam, validated for WB. https://www.abcam.com/veriblot-for-ip-detection-reagent-hrp-ab131366.html
-AF488-Goat anti mouse IgG, Validated by the company and cited in 667 publications. Statement from Thermofisher, validated for Flow Cyt, IP, IF, IHC. https://www.thermofisher.com/antibody/product/Goat-anti-Mouse-IgG-H-L-Cross-Adsorbed-Secondary-Antibody-Polyclonal/A-11001
-AF647-Goat anti Rat IgG, Validated by the company and cited in 20 publications. Statement from Thermofisher, validated for Flow Cyt, IP, IF, IHC, WB. https://www.thermofisher.com/antibody/product/Goat-anti-Rat-IgG-H-L-Cross-Adsorbed-Secondary-Antibody-Polyclonal/A-21247
-Qdot655-Goat anti rabbit IgG, Validated by the company and cited in 9 publications. Statement from Thermofisher, validated for IP, IF, IHC, WB. https://www.thermofisher.com/antibody/product/Goat-anti-Rabbit-IgG-H-L-Secondary-Antibody-Polyclonal/Q11422MP
-AF633-Goat anti Rat IgG, Validated by the company and cited in 14 publications. Statement from Thermofisher, validated for IP, IF, IHC. https://www.thermofisher.com/antibody/product/Goat-anti-Rat-IgG-H-L-Cross-Adsorbed-Secondary-Antibody-Polyclonal/A-21094
-Rabbit anti-FANCM antibody, Validated by the company and cited in 1 publication. Statement from Bethyl, validated for IP, WB. https://www.bethyl.com/search
-Rabbit anti-DONSON antibody, Validated by the company. Statement from Sigma, validated for IF, IHC, WB. https://www.sigmaaldrich.com/catalog/product/sigma/hpa049033?lang=en&region=US
-Mouse anti-H3K4me3, Validated by the company and cited in many publications, such as Proc Natl Acad Sci U S A. 2016 Feb23;113(8):2092-7 PMID:26862167. Statement from Absolute, validated for CHIP, ChIP-seq, IP, IP-MS, WB, ELISA, IF. https://absoluteantibody.com/product/anti-h3k4me3-304m3-b/
-Mouse anti-H3K9me3, Validated by the company and cited in many publications, such as Proc Natl Acad Sci U S A. 2016 Feb 23;113(8):2092-7 PMID:26862167. Statement from Absolute, validated for CHIP, ChIP-seq, IP, IP-MS, WB, ELISA, IF. https://absoluteantibody.com/product/anti-h3k9me3-309m3-b/
-Duolink® in situ oligonucleotide PLA probe PLUS, Validated by the company, cited in Molecular Cell 52(3):434-46. Statement from Sigma, validated for IF. https://www.sigmaaldrich.com/catalog/product/sigma/duo92002?lang=en&region=US
-Duolink® in situ oligonucleotide PLA probe MINUS, Validated by the company, cited in Molecular Cell 52(3):434-46. Statement from Sigma, validated for IF. https://www.sigmaaldrich.com/catalog/search?term=DUO92004&interface=All&N=0&mode=match%20partialmax&lang=en&region=US&focus=product

Eukaryotic cell lines

Policy information about cell lines

Cell line source(s)

HeLa (ATCC® CCL-2™) bought from ATCC.
hTERT RPE-1 (ATCC® CRL-4000™) bought from ATCC.
Hela/GFP and Hela/GFP-DONSON: HeLa-Flp-In T-REx host cells stably transfected with pcDNA5/FRT/TO-EGFP (vector only or expressing EGFP–DONSON). Constructed in Stewart laboratory (Reynolds et al. 2017).
DONSON P9: hTERT immortalized fibroblasts from patient 9, stably transduced with pMSCV-vector only or pMSCV-DONSON Constructed in Stewart laboratory (Reynolds et al. 2017).

Authentication

HeLa (ATCC® CCL-2™, https://www.atcc.org/products/all/CCL-2.aspx#characteristics), hTERT RPE-1 (ATCC® CRL-4000™, https://www.atcc.org/Products/All/CRL-4000.aspx#characteristics) were authenticated by ATCC.

Primary fibroblasts were derived from a skin biopsy taken from individual patients carrying biallelic mutations in DONSON previously identified by whole exome sequencing. DONSON mutations were validated by Sanger sequencing of gDNA and/or cDNA derived from the individual fibroblast cell lines. Reduced DONSON protein stability was verified by Western blotting of extracts derived from the individual patient-derived fibroblast cell lines. Immortalized complemented fibroblast cell lines were generated by transduction of the individual primary fibroblast cell lines with lentiviruses expressing hTERT and WT DONSON or an empty vector. Expression of exogenous DONSON was verified by Western blotting (Reynolds et al., 2017). Hela/GFP-DONSON (described by Reynolds et al., 2017) was authenticated the day of each experiment by verifying that the

	percentage of GFP(+) cells was greater than 90% by fluorescence microscopy, and saving an aliquot to confirm expression of GFP-DONSON of the expected molecular weight by western blot.
Mycoplasma contamination	All cell lines tested negative for Mycoplasma contamination. We regularly check all cell lines used in our laboratory with the Lonza MycoAlert Mycoplasma Detection Kit.
Commonly misidentified lines (See ICLAC register)	No commonly misidentified cell lines were used.

ChIP-seq

Data deposition

☒ Confirm that both raw and final processed data have been deposited in a public database such as GEO.

☒ Confirm that you have deposited or provided access to graph files (e.g. BED files) for the called peaks.

Data access links *May remain private before publication.*	Raw and processed data for FANCM and GFP-DONSON ChIp-seq experiments are deposited in GEO database under accession number GSE150550. Data access link: https://www.ncbi.nlm.nih.gov/geo/query/acc.cgi?acc=GSE150550
Files in database submission	Raw data files<br>NT_Input_index_20_S87_R1.fastq.gz<br>NT_Input_index_11_S86_R1.fastq.gz<br>NT_IP3_FANCM_index_1_S81_R1.fastq.gz<br>NT_IP3_FANCM_index_15_S80_R1.fastq.gz<br>HeLa_NT_Input_S1_R1_001.fastq.gz<br>HeLa_NT_Input_S1_R2_001.fastq.gz<br>HeLa_NT_GFP_S2_R1_001.fastq.gz<br>HeLa_NT_GFP_S2_R2_001.fastq.gz<br>Processed data files<br>NT_IP3_FANCM_Input_bwCompare_log2.bw<br>NT_IP3_FANCM_Input_Repl2_bwCompare_log2.bw<br>Hela_NT_GFP_Input_bwCompare_log2.bw<br>Hela_eigenvector_HiC.hg38.bedgraph
Genome browser session (e.g. UCSC)	No longer applicable

Methodology

Replicates	We performed two biological replicates. The log2 ratio between the ChIP-seq and the Input, as well as the ChIP-seq coverage of RT quantiles and Hi-C eigenvector quantiles of the two replicates agree with each other, as exemplified in Source data for Figure 6.
Sequencing depth	For the FANCM ChIP-seq experiment, single-end, 75 bp reads were collected, 144-172 million reads per sample were obtained and 140-171 million uniquely mapped reads were present. For the GFP-DONSON ChIP-seq experiment, paired-end, 75 bp reads were collected, 169-207 million reads per sample were obtained and 168-206 million uniquely mapped reads were present.
Antibodies	GFP Trap, Chromotek, Cat# gta-20, 25 µl per reaction, validated for IP, CHIP (Tian W. et al., 2019, NAR 47(10): 5436-5448), RRID: AB_2827592<br>Mouse anti FANCM, Sigma, Cat# SAB1407805, 1 in 100, validated for IP, WB, IF, RRID: AB_10760703
Peak calling parameters	No peak calling was done. Sequencing reads were aligned to the human hg38 reference genome using BWA mem 0.7.17. Duplicates were marked with Picard2.9.2 MarkDuplicates. Representative profiles from different chromosomes, comparing FANCM and GFP-DONSON distribution (enrichment = log2(ChIP/input)) in Hela cells stably expressing GFP-DONSON, to replication timing (RT = log2(Early/Late)) in Hela cells and A/B compartments as defined by the eigenvector calculated from Hi-C data from Hela cells, are shown in Figure 6a and Supplementary Fig. 6.
Data quality	No peak calling was done. The log2 ratio between FANCM or GFP-DONSON ChIP-seq and the Input was computed using Deeptools BigWigCompare of the corresponding RPKM normalized BigWig files. RT scores for FANCM and GFP-DONSON enriched genomic windows and ChIP-seq coverage of RT quantiles and Hi-C eigenvector quantiles is described in detail in Materials and Methods.
Software	Fastqc(0.11.9) for CHIp-seq data quality control<br>BWA v. 0.7.17, the mem algorithm was used to align each dataset to the hg38 human reference genome.<br>Picard v.2.9.2, to mark duplicates<br>SAMtools v.1.9, CHIP-seq analysis<br>BEDtools v.2.27.1, CHIP-seq analysis<br>DeepTools v.3.0.1, to compute the log2 ration between FANCM or GFP-DONSON ChIp-seq and the Input performing BigWigCompare of the corresponding RPKM normalized BigWig files.<br>Juicer v.1.5.6, to calculate the eigen vector from Hi-C data downloaded from NCBI database.<br>R v.3.5.2, CHIP-seq analysis, RT quantile analysis and graphs<br>UCSC toolkit v.388, to visualize RT, Hi-C eigen vector and log2 ratios of FANCM and GFP-DONSON Chip-seq and Input.

Flow Cytometry

Plots

Confirm that:

☒ The axis labels state the marker and fluorochrome used (e.g. CD4-FITC).

☒ The axis scales are clearly visible. Include numbers along axes only for bottom left plot of group (a 'group' is an analysis of identical markers).

☒ All plots are contour plots with outliers or pseudocolor plots.

☒ A numerical value for number of cells or percentage (with statistics) is provided.

Methodology

Sample preparation	GFP-DONSON cells were grown in culture and transfected with non-targeting siRNA, siDONSON, or siDONSON and siFANCM at day 1 and day 2. 72hr latter treated with Dig-TMP/UVA, followed by incubating with CldU and IdU. Cells were washed with PBS and digested by StemPro Accutase Cell Dissociation Reagent (Gibco, A11105-01), and washed with cold PBS, then incubated with Hoechst 33342 for 30 min at room temperature. Cells were suspended in 5% FBS HEPES buffer followed by sorting.
Instrument	Beckman Coulter moFlo XDP
Software	Data was analyzed with Summit V5.5.0.16880 software.
Cell population abundance	GFP-DONSON cells were grown in culture, detached by trypsinization and 100,000 cells/sample were analyzed. Full cell populations were analyzed without post-sort fractions.
Gating strategy	Gating was as follows: (1) Forward (FSC) and Side Scatter (SSC) to exclude debris, (2) FSC-A and FSC-H for doublet discrimination, (3) cells stained with Hoechst 33342, excited by ultraviolet light 355 nm, with an emission at 405 nm were gated based on DNA content into gate [R3] spanning late G1 and early S phase, and gate [R4] including cells spanning late S phase and G2. Gating example figures provided in Source Data for Figure 3b.<br>Sorted cells used in PLA experiments were classified as early or late S phase according to their characteristic PCNA staining pattern as described in Figure 3b, and widely accepted in the literature (Chagin V.O. et al, 2016, Nature Comm. 7:11231).

☒ Tick this box to confirm that a figure exemplifying the gating strategy is provided in the Supplementary Information.
